# Metabolomics reveals key biomarkers for ischemic stroke: a systematic review of emerging evidence

**DOI:** 10.3389/fneur.2025.1630390

**Published:** 2025-08-08

**Authors:** Lu Ding, Meiling Zhang, Baochao Fan, Fuyuan Deng, Zhenyuan Li, Yixuan Han, Yifan Wu, Jingchun Zeng, Liming Lu

**Affiliations:** ^1^Department of Traditional Chinese Medicine, The First Affiliated Hospital, Zhejiang University School of Medicine, Hangzhou, China; ^2^Clinical Research and Big Data Laboratory, South China Research Center for Acupuncture and Moxibustion, Medical College of Acu-Moxi and Rehabilitation, Guangzhou University of Chinese Medicine, Guangzhou, China; ^3^Medical Department, Zhejiang Provincial Hospital of Chinese Medicine, Hangzhou, China; ^4^School of European Languages and Cultures, Beijing Foreign Studies University, Beijing, China; ^5^College of Physical Education and Public Health, Guangzhou University of Chinese Medicine, Guangzhou, China; ^6^Department of Rehabilitation, The First Affiliated Hospital, Guangzhou University of Chinese Medicine, Guangzhou, China; ^7^ShenShan Hospital, The First Affiliated Hospital of Guangzhou University of Chinese Medicine, Shanwei, China

**Keywords:** ischemic stroke, metabolomics, biomarkers, diagnosis, risk prediction, prognosis

## Abstract

**Objective:**

To systematically collate and evaluate metabolomics-based biomarkers of ischemic stroke (IS) to guide clinical diagnosis and treatment.

**Methods:**

Comprehensive literature searches were conducted in PubMed, Embase, and Web of Science using “IS” and “metabolomics” as core keywords, covering publications up through February 2024. Any original metabolomic research related to IS was selected. Key information such as study demographics, study type, objectives, metabolomic analysis methods, and main findings were extracted and analyzed. Frequently mentioned metabolites were subjected to enrichment analysis using the MetaboAnalyst 6.0 platform.

**Results:**

A total of 51 studies were included. Quality assessment revealed that 54.8% of the diagnostic studies and 69.2% of the prognostic studies were high-quality, with most controlling for confounding factors. Metabolite analysis revealed associations between decreased proline, isoleucine, valine, and alanine levels with IS. Increased tyrosine, glutamine, phenylalanine, sphingomyelin, glutamate, lactate and glucose, and decreased LysoPC (18:2), histidine, and methionine levels were linked to IS onset. Specific metabolite combinations, such as serine, isoleucine, betaine, PC (5:0/5:0), and LysoPE (18:2), showed high precision in predicting acute ischemic stroke (AIS) (training set AUC = 0.988, test set AUC = 0.971). Glycine-serine-threonine and valine-leucine-isoleucine pathways were significant in diagnosing IS and AIS, and in differentiating ischemic and hemorrhagic strokes, as well as identifying post-stroke depression and cognitive impairment.

**Conclusion:**

This study confirms the potential diagnostic and prognostic value of changes in amino acids and lipids, as well as other metabolites and metabolic pathways, in IS. These findings highlight the promise of metabolomics in IS diagnosis, differential diagnosis, risk assessment, and complication identification. However, further validation is needed due to the varying quality of the included studies.

**Systematic review registration:**

https://www.crd.york.ac.uk/PROSPERO/#myprospero, identifier CRD42022335505.

## Introduction

Stroke, characterized by its high recurrence and disability rates, has become the second leading cause of death in humans (1). Researchers have been seeking biomarkers to improve the diagnosis of stroke and identify its etiology. Accurate diagnosis of ischemic stroke (IS) and its causes is crucial for selecting the best treatment and prevention strategies; however, the diagnosis and classification of IS still primarily dependent on clinical history and neuroimaging techniques, which are not fully implemented in all levels of hospitals, and are both costly and time-consuming (2, 3). In acute cases, particularly when clinical symptoms are mild, distinguishing IS from other neurological diseases and determining the cause of stroke is challenging. Therefore, new biomarkers for the rapid and accurate prediction, diagnosis, and classification of IS could play a positive role in elucidating the pathophysiological mechanisms of IS and promoting management in IS patients.

Due to the presence of the blood–brain barrier (BBB), it is difficult for large molecules to be released from the brain into the bloodstream. Thus, researchers struggle to identify specific and sensitive metabolites in IS patients with conventional detection methods (4). However, with the advancement of biological systems, it is possible to identify specific small molecule biomarkers in IS patients using emerging technologies, and to determine potential causes (5, 6). Metabolomics, a key branch of systems biology, is an effective method for revealing biomolecular phenotypes. It has shown great potential in identifying changes in small molecule metabolites across various diseases, including the discovery of biomarkers and mechanistic studies (7), particularly in the field of cardiovascular and cerebrovascular diseases (8). Metabolomic approaches offer a unique perspective on metabolic changes and potential biomarkers during pathological processes, and studies have shown that changes in metabolite levels in biological fluids may be consistent with metabolic changes in the brain (9). IS is a major disease with poor prognosis, characterized by complex pathological mechanisms involving alterations in multiple metabolic pathways. Therefore, a thorough investigation into the application of metabolomics in IS is of great significance for early diagnosis, treatment, and prognosis assessment of the disease.

Recent studies have utilized metabolomics to explore serum, plasma, and brain tissue samples from humans and animals with IS, identifying metabolites related to stroke pathogenesis. For instance, specific lipid metabolism changes are closely associated with IS onset (10). Additionally, abnormalities in amino acid metabolism related to energy metabolism and excitotoxicity are significant markers for IS onset and may play a crucial role in post-stroke recovery (11). These findings offer new perspectives on stroke biomarkers and have profound implications for clinical diagnosis and treatment strategies. Despite the increasing use of metabolomics in IS, the methodology and quality of existing studies are uneven, leading to gaps in the knowledge of IS biomarkers. Moreover, there is a lack of systematic integrated assessment of these studies for a comprehensive understanding of IS biomarkers.

The aim of this systematic review is to comprehensively summarize and analyze metabolomics-based biomarkers for IS, including those identified through biological samples such as serum, plasma, urine, and brain tissue. By synthesizing data from studies that utilized these tissue types, we seek to identify reliable and representative biomarkers for IS, which could aid in early diagnosis, treatment selection, and monitoring. This review will focuses on evaluating the potential of these biomarkers in three key areas: the diagnosis of IS, the prognosis of patient outcomes, and the prediction of future stroke risks or related complications. The findings are intended to provide a clearer understanding of the role of metabolomics in IS and contribute to the development of clinically relevant biomarkers that could inform both diagnosis and therapeutic strategies.

## Methods

This system review has been registered on PROSPERO (CRD42022335505).

### Literature search

We systematically searched the PubMed, Embase, and Web of Science (WOS) databases for literature published up through February 1, 2024. Keywords were combined in a mesh words plus free words strategy to ensure comprehensive literature retrieval, with initial search terms being (“metabolomics” or “metabolomics spectrum” or “metabolic characteristics” or “metabolic biomarkers” or “metabolite spectrum”) and (“stroke” or “cerebral infarction” or “cerebral ischemia” or “cerebrovascular disease attack” or “acute cerebrovascular accident” or “acute cerebrovascular disease”). Two researchers (LD and ZL) independently conducted the searches, with any disagreements or uncertainties resolved through discussion with a third researcher (LL). (Specific search strategies can be found in Supplement 1).

### Inclusion and exclusion criteria

(1) Inclusion criteria.

①Research population: Includes patients diagnosed with IS by specific diagnostic criteria [World Health Organization (WHO), American Heart Association/American Stroke Association (AHA/ASA) guidelines, etc.] (12).
②Research types: Observational studies such as case-control studies, cohort studies and cross-sectional studies.
③Research content: The application of metabolomics in diagnosis, prognosis evaluation, etc.
④Data type: Human research data, including clinical data and biomarker data.
⑤Language of publication: English or Chinese.
(2) Exclusion criteria.

①Animal experiments, drug trials, reviews, systematic reviews/meta-analyses, conference abstracts, review articles, case reports, expert opinions, consensus guidelines.
②Studies which have yet to be published or which have incomplete data.
③Studies with repeated publications or overlapping data.
④Patients with subarachnoid hemorrhage or cerebral hemorrhage caused by trauma.
⑤The full text of the study could not be obtained by contacting the original author.

### Metabolomic measurement standards

To ensure the reliability and comparability of metabolomic data, only studies that adhered to strict methodological standards were included. Studies were required to use validated analytical platforms, including liquid chromatography-mass spectrometry (LC-MS), gas chromatography-mass spectrometry (GC-MS), and nuclear magnetic resonance (NMR), to ensure consistent and reproducible results, and clear reporting of sampling methods, sample processing, and metabolomic analysis protocols. Specific quality control measures, such as the use of internal standards and calibration checks, were required to ensure that the results were consistent and reproducible across studies. Studies lacking these critical methodological details were excluded.

### Data extraction

After reading the full texts and Supplementary material, two researchers (LD and MZ) independently extracted the following information from the included studies: basic literature details, study population, group settings, study types, sample size, IS sampling time window, study objectives, sample types, technological platforms, targeted/non-targeted approach, potential metabolic biomarkers and their changes in direction. Two researchers cross-checked the final extraction results to improve the extraction accuracy. If any differences were found in the data extraction results, they were resolved through discussion or the ruling of a third researcher (LL). If a study included both exploratory and confirmatory cohorts, only data from the exploratory cohorts was extracted.

### Quality assessment

To assess the methodological quality of studies related to the metabolic profiling of IS in clinical diagnosis and complication identification, we employed the Quality Assessment of Diagnostic Accuracy Studies for omics (QUADOMICS) tool. This tool is an Adaptation of the Quality Assessment of Diagnostic Accuracy Studies (QUADAS) tool for assessing the quality of diagnostic accuracy studies. It is made up primarily of 16 items: whether a representative patient population was appropriately selected; how the omics studies were conducted or if clear testing standards were present; whether appropriate reference standards were used and how they were compared with the omics test results; whether the process from patient inclusion to results acquisition was appropriate and whether there were any potential delays that could have affected diagnostic accuracy; whether the interpretation of the results employed blinding; how uncertain or ambiguous diagnostic results were handled; whether patient dropouts were reported in the study and assessing whether these dropouts could have influenced the outcomes; and whether the reporting of the study results was complete and whether there were any signs of selective reporting (13). Additionally, for prognosis or risk prediction type studies, we used the Quality In Prognosis Studies (QUIPS) tool. This tool employs a six-point rating scale to assess the quality of metabolomics studies related to predicting IS risk and recurrence, covering criteria such as the representativeness of the study sample, study dropout rates, prognostic factor measurement, outcome measurement, adjustments for confounding factors, and statistical analysis reporting (14). Higher scores on these tools indicate higher quality research, with a study considered high quality if it meets more than 80% of the items, moderate quality if it meets 50 to 80% of the items, and low quality if it meets less than 50% of the items. The thresholds used for quality classification were based on established guidelines from previous studies (13, 14). These criteria are relevant as they allow for the systematic categorization of study quality, ensuring a more rigorous and consistent evaluation of the studies included in this review.

### Statistical analysis

To gain a deeper understanding of the metabolic biomarkers and pathways associated with IS, this study includes statistical analysis on the frequency of biomarkers repeatedly reported in the included studies. Initially, data concerning these variables from all studies were collected, followed by descriptive statistical analysis using RStudio version 4.1.3, including calculations of frequencies and percentages, and visual presentations of these data with bar charts to show the usage frequency of different analytical platforms and biological samples. Metabolic pathway analysis was conducted using MetaboAnalyst 6.0, an integrated platform for metabolomics data analysis available online at http://www.metaboanalyst.ca/. This platform was used for enrichment analysis to identify any significantly altered metabolic pathways in the included studies, aiding in the revelation of potential biological mechanisms (15). Additionally, topology analysis was conducted to evaluate the significance and centrality of the metabolites within the metabolic network, helping to identify any potential key metabolic biomarkers or regulatory nodes (16). The bubble chart of metabolic pathway enrichment displays the log(*p*) value on the vertical axis and the rich factor, calculated based on topology analysis, on the horizontal axis. The rich factor represents the proportion of differential metabolites annotated in a given pathway.

## Results

### Characteristics of the included studies

**Figure 1:**
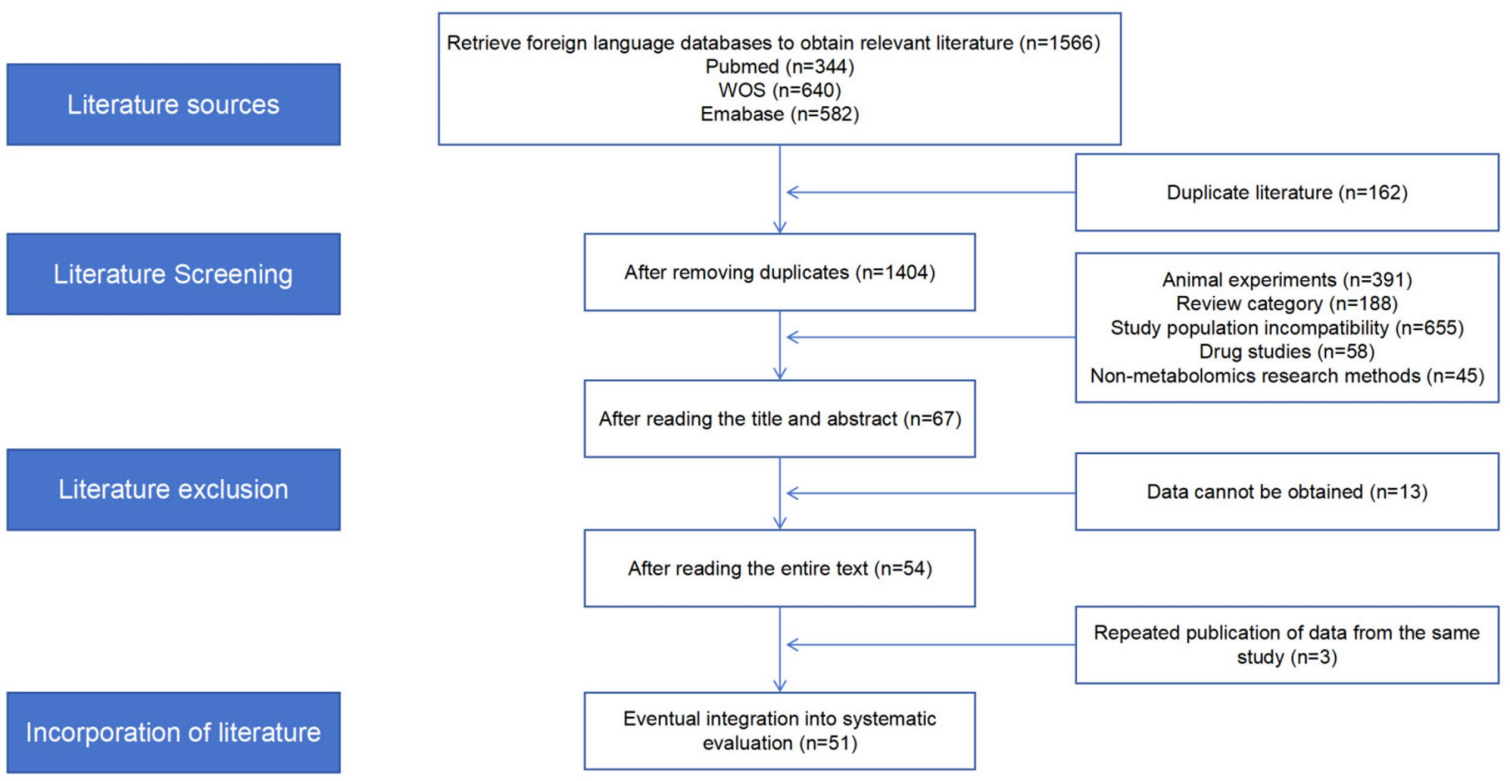
Flow chart of the PRISMA research process.

In this study, a total of 1,566 research articles were retrieved through three electronic databases. After deduplication, 162 studies were excluded. Following a review of the abstracts, 1,377 studies were excluded. Thirteen studies were further excluded after full-text review due to incomplete data or the inability to obtain complete data. Upon thorough examination of the full texts, three studies were found to be consistent, but to be duplicate publications, and were therefore excluded. Fifty-one relevant studies were included in the final systematic review. The research process is depicted in the flowchart in Figure 1.

The 51 studies included a total of 20,971 participants, with individual study sizes ranging from 40 to 3,904 participants. Among these, 32 studies (62.7%) were conducted in China, 5 (9.8%) in South Korea, 6 (11.8%) in the USA, 2 (3.9%) in Brazil, 2 (3.9%) in Germany, and the remaining 4 (7.8%) in the UK, France, India, and Portugal, respectively. Among all the studies, 43 (84.3%) used a case-control design, 3 (5.9%) used a prospective cohort design, 2 (3.9%) used a retrospective cohort design, 2 (3.9%) combined case-control and cohort designs, and 1 (2.0%) employed a cross-sectional design.

The majority of the studies (*n* = 30, 59%) focused on differentiating between stroke patients and healthy or matched controls. Two studies (3.9%) distinguished between ischemic and hemorrhagic stroke, six studies (11.8%) identified post-stroke depression (PSD), and one study (2.0%) recognized post-stroke cognitive impairments (PSCI). Thirty-eight studies (74.5%) were applicable to clinical diagnosis, 7 (13.7%) to the identification of complications, 7 (13.7%) to risk prediction, 2 (3.9%) to recurrence prediction, and 4 (7.8%) to outcome prognosis assessment.

Analytical platforms included LC-MS in 30 studies (58.8%), GC-MS in eight studies (15.7%), dual gas-liquid chromatography-mass spectrometry in two studies (3.9%) and NMR in nine studies (17.6%). Two studies (3.9%) mentioned mass spectrometry (MS) analysis without specifying the platform. Thirty-seven studies (72.5%) were non-targeted, 9 (17.6%) were targeted, 2 (3.9%) combined targeted and non-targeted approaches, and 3 (5.9%) did not report on whether they were targeted.

In terms of sample type, 45 studies (88.2%) used blood, 4 (7.8%) used urine, 2 (3.9%) used both blood and urine, and 1 (2.0%) used blood, urine, and feces. (See Supplement 2 for a summary of the study characteristics).

### Quality evaluation

In the 42 diagnostic studies, 23 (54.8%) achieved a total score exceeding 13 points (a perfect score was 16 points), qualifying them as high-quality studies. Only 5 studies (11.9%), described intermediate test results and included overfitting checks (17–21). Among the 13 prognostic studies, 9 (69.2%) scored above 5 points (a full score was 6 points), and 10 (76.9%) controlled for potential confounding factors with appropriate methods; six studies (46.2%) reported details of subject loss to control for potential bias (11, 22–26). (See Supplement 3 for quality evaluation results).

### Overview of metabolic biomarkers related to IS

Compared to the normal control group, eight metabolomic studies (19, 27–33) reported a range of differential metabolites related to acute ischemic stroke (AIS), including isoleucine, inosine, acetoacetate, and β-hydroxybutyrate. Twenty-three studies (17, 21, 24, 26, 34–51) reported differential changes in metabolites such as amino acids and their derivatives, lactate, uric acid, choline, carnitine, sphingolipids, and glucose in IS patients compared to controls. Two studies (33, 37) identified valine, leucine, tyrosine, and 3-hydroxybutyrylcarnitine as differential metabolites distinguishing ischemic from hemorrhagic stroke. Six studies (52–57) associated metabolites such as palmitic acid; oleic acid, lactate, and amino acids with post-stroke depression (PSD), and metabolites such as lysophosphatidylcholine (LysoPC), carnitine, amino acids, uric acid, and sphingomyelin with PSCI (58). Six studies (22, 25, 43, 59–61) suggested that a range of fatty acids, betaine, ceramides, choline, and amino acids could serve as predictive metabolites for IS risk. Metabolites such as glutamic acid, arginine, proline, glycyrrhetinic acid, octylcarnitine, and formylcarnitine can be used as predictors of IS recurrence and prognosis (11, 18, 23, 26, 35, 62).

### High-frequency metabolic biomarkers related to IS

**Figure 2:**
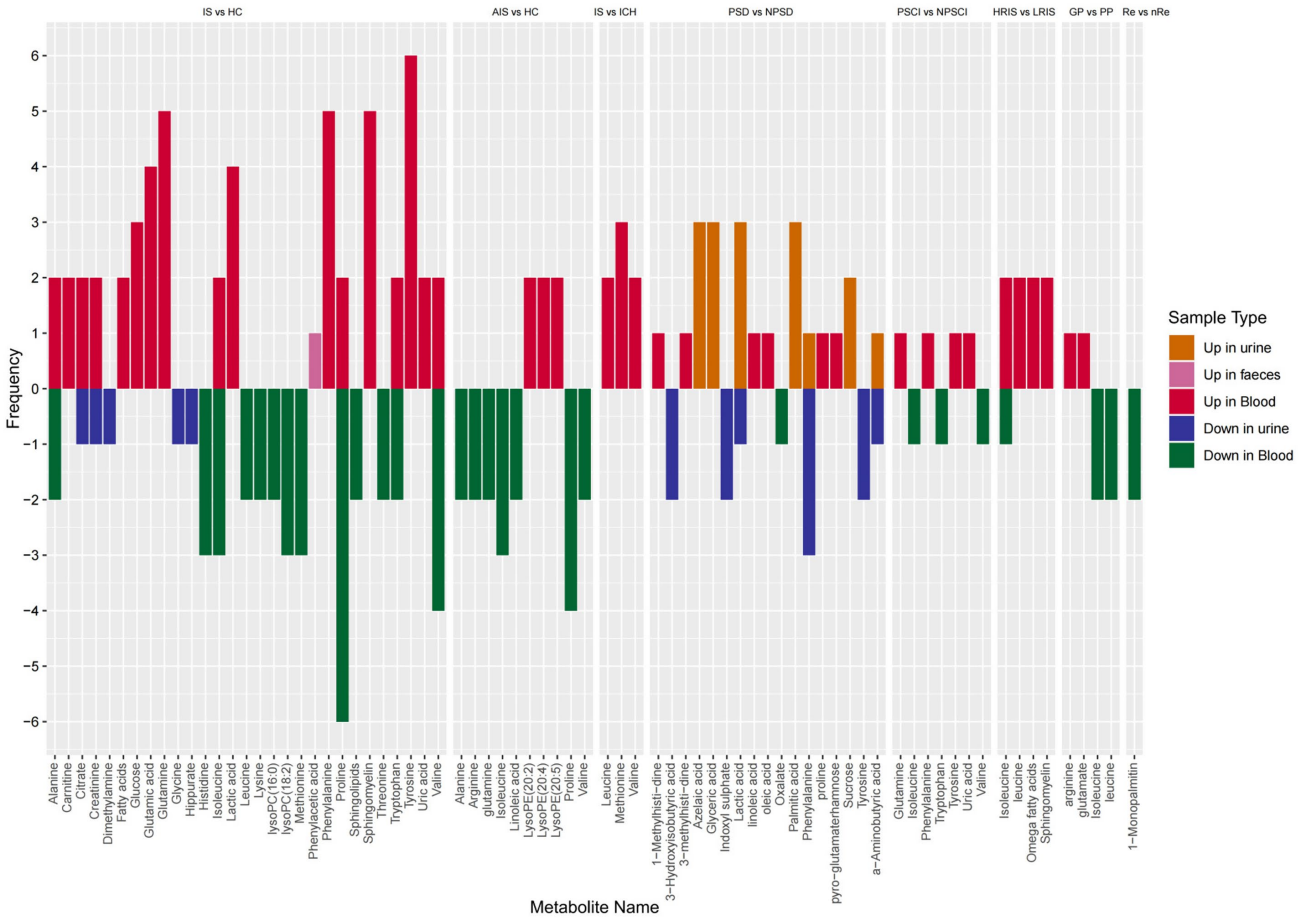
Summary of high-frequency metabolic biomarkers related to IS.

To minimize the influence of potential confounding factors, in this study, we selected studies with a QUADOMICS score ≥12 points or QUIPS score ≥4. Five points for a summary of metabolite frequencies, showing which potential metabolites are related to IS in different metabolomics studies, and visually presenting the results in stacked bar charts (as shown in Figure 2).

From the chart, it is evident that the main upregulated metabolites in IS compared to the normal control group are tyrosine, glutamine, phenylalanine, sphingolipids, glutamate, lactate and glucose; the main downregulated metabolites are proline, valine, histidine, isoleucine, LysoPC (18:2) and methionine; additionally, alanine, citrate, creatine, isoleucine, tryptophan and valine exhibited both upregulated and downregulated trends.

Compared to the normal control group, the main upregulated metabolites in AIS include three ratios of lysophosphatidylethanolamine (LysoPE) (20:2) (20:4) (20:5), with the main downregulated metabolites being proline, isoleucine, glutamine, arginine, valine, alanine and linoleic acid.

Compared to hemorrhagic stroke, IS showed elevated levels of methionine, leucine and valine. In PSD compared to stroke patients, upregulated metabolites include palmitic acid, glycerate, lactate, azelaic acid and sucrose, while downregulated metabolites include indole sulfate, phenylalanine and tyrosine, with both palmitic acid and tyrosine showing consistent trends in both urine and blood samples. However, phenylalanine and α-aminobutyrate exhibited opposing trends in blood and urine samples, especially phenylalanine, which showed both upregulated and downregulated trends in urine samples.

For PSCI patients, the main upregulated metabolites in blood are glutamine, uric acid, tyrosine and phenylalanine, with downregulated metabolites including tryptophan, isoleucine and valine. Predicting high and low IS risk primarily relates to the upregulation of omega fatty acids, sphingolipids, isoleucine and leucine, although isoleucine shows variability in both directions. Prognosis prediction for IS is primarily related to the upregulation of glutamate and arginine, and the downregulation of isoleucine and leucine. Due to limited studies on IS recurrence, only the upregulation of 1-monopalmitin was found.

Overall, isoleucine and proline consistently show a marked downregulation in various diagnostic and predictive factors of IS, while tyrosine, phenylalanine, valine and arginine exhibit opposite trends.

### The predictive potential of metabolic biomarker combinations related to IS

Among the included studies, 25 examined the potential of metabolic biomarker combinations for predicting and diagnosing IS, as summarized in Table 1: the combination of serine, isoleucine, betaine, PC (5:0/5:0), and LysoPE (18:2) showed the highest accuracy in predicting AIS in the test set (19) (training set AUC = 0.988, test set AUC = 0.971); 1-methylhistidine, 3-methylhistidine, LDL CH_3_-(CH_2_)n, phenylalanine, and tyrosine were identified as potential metabolic biomarker combinations for recognizing PSD (54), exhibiting the highest accuracy in the test set (AUC = 0.969); glutamine, xanthine, and LysoPC (18:2) as potential combinations for identifying PSCI (58) (training set AUC = 0.96, test set AUC = 0.87); a combination of 12 metabolites including 2-methyl-1-pyrroline, D-pipecolinic acid, and DL-dihydrosphingosine was used for predicting the risk levels of IS (59) (AUC = 0.848); LysoPC (20:4), (16:0) as a potential combination for predicting IS recurrence (23) (AUC = 0.74); and proline, glutamate, and arginine as potential combinations for predicting the outcome of IS (11) (training set AUC = 0.952, test set AUC = 0.835).

**Table 1:** Classification/predictive potential of combined biomarkers.

Literature references	Combination of potential metabolic markers	Training set AUC	Training set sample size	Test set AUC	Test set sample size	Object differentiation
Balasubramanian et al. (47)	N6-Acetyllysine, methionine sulfoxide (MetSO), sucrose/lactose/trehalose, glucuronate			0.7	167	IS vs. HC
Chen et al. (52)	3-Hydroxyphenylacetic acid, tyrosine, phenylalanine, sucrose, palmitic acid, glyceric acid, azelaic acid, α-aminobutyric acid	0.942	212	0.868	112	PSD vs. none-PSD
Guo et al. (59)	9-Cis-retinal, DL-indole-3-lactic acid, methyl ricinoleate, (2S)-OMPT, 2-methyl-1-pyrroline, D-pipecolinic acid, 7,7-dimethyl-5,8-eicosadienoic acid, PC(15:0/0:0)[U], DL-dihydrosphingosine, bufexamic acid,10-hydroxy-2-decenoic acid, L-thyroxine	0.848	132			High risk IS vs. low risk IS
Hu et al. (37)	Asparagine, tiglylcarnitine, (arginine)/(ornithine), (valine)/(phenylalanine), (free carnitine + acetylcarni tine + propionylcarnitine + palmitoylcarnitine + petrose linic carnitine)/(citrulline)	0.873	202			IS vs. HC
Hu et al. (54)	1-Methylhistidine, 3-methylhistidine, LDL CH_3_-(CH_2_)n, phenylalanine, tyrosine			0.969	22	PSD vs. NPSD
Jové et al. (23)	Lysophosphatidylcholine 20:4, lysophosphatidylcholine 16:0			0.74	162	SR vs. non-SR
Kong et al. (24)	Vanylglycol, L-2,4-diaminobutyric acid, 2-phenylglycine		55			CI vs. HC
Lee et al. (83)	Lysine, serine, threonine, putrescine, kynurenine, and lysophos phatidylcholine acyl C16:0	0.895	193	0.811	123	LAA vs. CE
Liu et al. (58)	Glutamine, kynurenine, and LysoPC (18:2)	0.96	40	0.87	20	PSCI vs. none-PSCI
Liu et al. (19)	Serine, isoleucine, betaine, PC(5:0/5:0), lysoPE (18:2)	0.988	80	0.971	49	AIS vs. HC
Qi et al. (30)	Argininosuccinic acid, β-D-glucosamine, glycerophosphocholine, L-abrine, L-pipecolic acid	0.919	44			AIS vs. HC
Sun et al. (20)	Carnitine C18:1, arginine/ornithine, 3-hydroxylbutyrylcarnitine, arginine	0.89	84			AIS vs. vertigo
Sun et al. (39)	Uric acid, sphinganine, adrenoyl ethanolamide	0.941	60			IS vs. HC
Tiedt et al. (44)	Asymmetrical imethylarginine, symmetrical dimethylarginine, pregnenolone sulfate, adenosine			0.9	210	IS vs. SMs
Wang et al. (40)	Tyrosine, lactate, tryptophan	0.917	69			IS vs. control
Wang et al. (11)	Proline, glutamic acid, arginine	0.952	40	0.835	40	GP vs. PP
Wu et al. (62)	Glycocholic acid	0.68	100			GP vs. PP
Xiao et al. (55)	Lactate, phenylalanine, α-hydroxybutyrate, formate, arabinitol	0.946	150	0.946	96	PSD vs. NPSD
Xie et al. (56)	Palmitic acid, hydroxylamine, myristic acid, glyceric acid, lactic acid, tyrosine, azelaic acid	0.92	117	0.877	64	PSD vs. NPSD
Yang et al. (84)	Glucosylceramide (38:2), phosphatidylethanolamine (35:2), free fatty (16:1), triacylglycerol (56:5)	1	12	0.947	44	IS vs. HC
Yu et al. (46)	Phenylacetylglutamine (PAGln)	0.963	951	0.7	200	IS vs. HC
Yang et al. (51)	CerP (181/20:3), CerP (181/18:1), CerP (181/180), CerP (1811/16), SM (181/261), Cer (180/20:0)	0.953	56	0.894	56	Stroke vs. HC
Yu et al. (45)	Sphingosine-1-phosphate	0.701	96	0.738	80	GCC vs. PCC
Zhang et al. (57)	Azelaic acid, glyceric acid, pseudouridine, 5-hydroxyhexanoic acid, tyrosine, phenylalanine	0.961	218	0.954	167	PSD vs. NPSD
Zhou et al. (85)	SM (d18:0/14:0), PC (18:2(9Z,12Z))/18, lysoPC(P-18:0/0:0), 1-methylpyrroliniu, PC (18:0/P-18:0)	0.841	160	0.787	240	IS vs. HC

AUC, area under the curve; CerP, ceramide-1-phosphoceramide; SM, sphingolipids; PC, phosphatidylcholine; PG, phosphatidylglycerol; CE, cholesterol esters; LysoPC, lysophosphatidylcholine; LysoPE, lysophosphatidylethanolamine; AIS, acute ischemic stroke; IS, ischemic stroke; CI, cerebral infarction; HC, healthy control; GP, good prognosis; PP, poor prognosis; GCC, good collateral circulation; PCC, poor collateral circulation; SR, stroke recurrence; LAA, large artery atherosclerosis; CES, cardioembolic stroke; SMs, stroke mimics; PSD, post stroke depression; PSCI, post-stroke cognitive impairment.

### Summary of metabolic pathways related to IS

**Figure 3:**
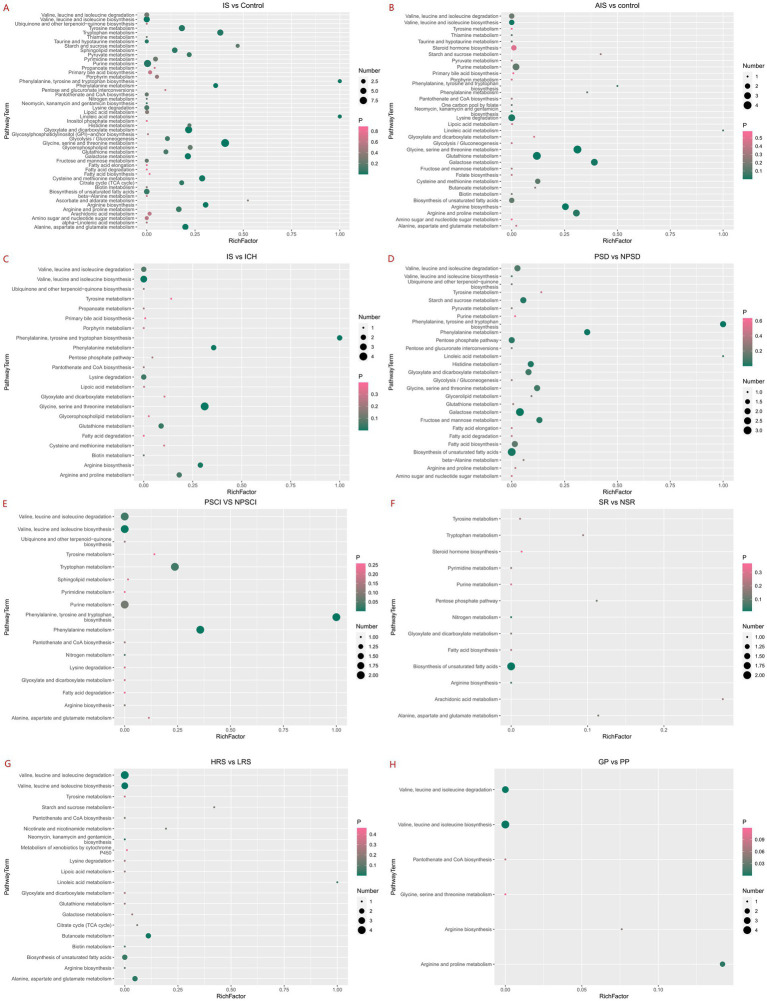
Enrichment of IS-related metabolic pathways. ^*^Bubble plot of metabolic pathway enrichment analysis showing significantly enriched pathways in ischemic stroke. Each bubble represents a metabolic pathway, with the size indicating the number of metabolites involved and the color reflecting the pathway’s enrichment score. The bubble chart of metabolic pathway enrichment displays the log(*p*) value on the vertical axis and the rich factor, calculated based on topology analysis, on the horizontal axis. The rich factor represents the proportion of differential metabolites annotated in a given pathway.

For IS compared to the control group, significant enrichment was observed in 14 metabolic pathways: glycine-serine-threonine metabolism, biosynthesis of valine-leucine-isoleucine, malate-dicarboxylate metabolism, arginine biosynthesis, phenylalanine metabolism, galactose metabolism, alanine-aspartate-glutamate metabolism, biosynthesis of phenylalanine-tyrosine-tryptophan, cysteine-methionine metabolism, linoleic acid metabolism, purine metabolism, tryptophan metabolism, tyrosine metabolism, and taurine and hypotaurine metabolism (Figure 3A).

In AIS compared to the control group, significant enrichment was observed in 7 metabolic pathways: glutathione metabolism, arginine biosynthesis, glycine-serine-threonine metabolism, biosynthesis of valine-leucine-isoleucine, galactose metabolism, lysine degradation and arginine-proline metabolism (Figure 3B).

For distinguishing ischemic from hemorrhagic strokes, significant enrichment was found in seven metabolic pathways: biosynthesis of valine-leucine-isoleucine, glycine-serine-threonine metabolism, biosynthesis of phenylalanine-tyrosine-tryptophan, phenylalanine metabolism, arginine biosynthesis, glutathione metabolism and lysine degradation (Figure 3C).

In identifying PSD, significant enrichment was noted in 8 pathways: biosynthesis of phenylalanine-tyrosine-tryptophan, phenylalanine metabolism, galactose metabolism, biosynthesis of unsaturated fatty acids, histidine metabolism, starch-sucrose metabolism, fructose-mannose metabolism, and the pentose phosphate pathway (Figure 3D).

For identifying PSCI, six pathways showed significant enrichment: biosynthesis of phenylalanine-tyrosine-tryptophan, biosynthesis and degradation of valine-leucine-isoleucine, phenylalanine metabolism, tryptophan metabolism and nitrogen metabolism (Figure 3E).

Only nitrogen metabolism and biosynthesis of unsaturated fatty acids were significantly enriched in predicting IS recurrence (Figure 3F). For predicting the risk of IS, significant enrichment was noted in seven pathways: biosynthesis and degradation of valine-leucine-isoleucine, butyrate metabolism, biosynthesis of neomycin-kanamycin-gentamicin, alanine-aspartate-glutamate metabolism, biosynthesis of unsaturated fatty acids and linoleic acid metabolism (Figure 3G). In predicting the outcome of IS, significant enrichment was observed in three pathways: biosynthesis and degradation of valine-leucine-isoleucine and arginine-proline metabolism (Figure 3H).

Details on the metabolic pathway names, matching status, *p*-values, and pathway impact are provided in Supplement 4.

## Discussion

### Principal findings

Timely delineation of stroke subtype, estimation of recurrence risk, and anticipation of post-stroke sequelae remain critical yet unmet needs in the management of IS (63, 64). Metabolomics, by delivering a high-resolution snapshot of low-molecular-weight metabolites through untargeted LC-MS or NMR platforms, can discover biomarkers beyond the reach of traditional assays (65). In this systematic review of 51 metabolomics studies, we identified a reproducible metabolic signature of IS involving alterations across amino acid, lipid, and energy metabolism pathways. The metabolites most consistently associated with IS were decreased isoleucine and proline levels, alongside elevated concentrations of tyrosine, phenylalanine, and sphingomyelin. Given the pathophysiological heterogeneity of IS, reliance on a single metabolite marker has limited clinical utility; thus, multi-marker panels demonstrated superior diagnostic performance (66). A five-marker panel—serine, isoleucine, betaine, phosphatidylcholine (5:0/5:0), and lysophosphatidylethanolamine (18:2)—achieved an area under the curve of 0.97 in an external test set for acute IS, illustrating the diagnostic gain obtained by integrating markers that reflect atherosclerosis, thrombosis, inflammation, and oxidative stress (3). Pathway enrichment highlighted glycine-serine-threonine metabolism and valine-leucine-isoleucine biosynthesis, implicating disturbed bioenergetics and branched-chain amino-acid catabolism as potential therapeutic targets. Finally, temporal stratification showed that the metabolic signature of the acute phase differs markedly from that of the recovery phase, underscoring the need to consider sampling window when deploying metabolomic biomarkers in both research and clinical practice.

### Comparison of key findings with previous research

Our findings align with and extend existing metabolomic evidence in ischemic stroke (IS). Similar to prior studies by Ke et al. (67), amino acids (e.g., isoleucine, glutamate) and lipid metabolites (LysoPC, PC) were repeatedly identified as key indicators linked to stroke pathogenesis. Alanine-aspartate-glutamate metabolism also emerged consistently across clinical and animal studies, reinforcing its biological relevance (68). Additionally, the prominent role of glycine-serine-threonine metabolism, involved in oxidative stress and inflammation after neuronal injury, corroborates previous mechanistic insights, highlighting potential therapeutic targets. Notably, we observed alterations in phenylalanine and tyrosine metabolism that were less consistently reported previously. The variability of phenylalanine across studies may reflect differences in patient characteristics, stroke severity, sample timing, and analytical methods, emphasizing the need for standardized protocols in future research. Its clinical utility for diagnosis or prognosis remains uncertain and requires further evaluation. Compared to earlier systematic reviews, our analysis integrates diagnostic, prognostic, and risk-prediction biomarkers across diverse sample types and methodologies. In contrast to the review by Zhang et al. (69), which lacked formal quality assessment, our rigorous appraisal using QUADOMICS and QUIPS, along with standardized pathway enrichment analysis, enhances comparability and reliability. Our broader inclusion criteria further improve the generalizability and robustness of the identified biomarkers.

### Clinical implications and mechanistic links of metabolomic biomarkers in IS

The metabolomic biomarkers identified in this review not only demonstrate potential clinical value for IS diagnosis, prognosis, and risk prediction, but also align closely with well-established pathophysiological mechanisms, reinforcing their translational validity. For instance, elevated glutamate—consistently identified across diagnostic and prognostic analyses—directly reflects neuronal excitotoxicity, a fundamental driver of ischemic neuronal damage (58). Excessive glutamate release during cerebral ischemia leads to neuronal calcium overload and cell death via NMDA receptor overstimulation, providing a robust biological rationale for its prognostic relevance in IS outcomes (70, 71). Similarly, observed alterations in phenylalanine and tyrosine metabolism likely indicate heightened oxidative stress and impaired phenylalanine hydroxylase (PAH) activity during acute ischemia (72, 73). Oxidative stress-induced reduction of PAH activity, mediated by tetrahydrobiopterin (BH4) deficiency, not only disrupts amino acid metabolism but also affects nitric oxide synthesis, endothelial function, and neurotransmitter availability—pathways closely associated with stroke severity and post-stroke complications such as cognitive impairment and depression (74). Reduced plasma proline levels, another consistent finding linked with poor functional outcomes, mechanistically correspond with heightened inflammatory responses and endothelial injury. Proline metabolism closely interacts with inflammatory cascades, leukocyte activation, and matrix metalloproteinase-9 (MMP-9) mediated blood–brain barrier disruption, processes critically implicated in secondary ischemic injury progression and hemorrhagic transformation risks (75, 76). Additionally, sphingolipid metabolism perturbations (particularly sphingomyelin elevation) identified herein are closely associated with endothelial dysfunction and compromised blood–brain barrier integrity. Sphingolipids regulate cell proliferation, apoptosis, inflammatory signaling, and endothelial permeability—all crucial pathways mediating stroke severity, recurrence risk, and complication development. Thus, biomarkers targeting sphingolipid alterations may directly reflect critical processes influencing stroke recovery trajectories and recurrence (77). These molecules act on various protein targets, including kinases, phosphatases, lipases, and other enzymes and membrane receptors, exerting diverse cellular functions (78, 79). In addition to these core pathways, branched-chain amino acids (BCAAs)—including valine, leucine, and isoleucine—emerged across multiple studies as significantly altered in IS patients. These amino acids are involved in mitochondrial energy metabolism, neurotransmitter biosynthesis, and redox homeostasis (80, 81). Decreased plasma BCAA levels have been associated with infarct size and greater disability, while multivariate models incorporating BCAAs have demonstrated discriminative value in distinguishing IS from TIA or non-stroke controls (35). However, while plasma-based metabolomics provides valuable insights, it may not fully reflect cerebral metabolic activity. Several animal studies have highlighted discordance between peripheral and central metabolite changes after ischemia-reperfusion injury. For example, methionine is consistently downregulated in plasma but upregulated in ischemic brain tissue, underscoring the tissue-specificity of metabolic responses (42, 82). These discrepancies caution against overinterpretation of blood-based biomarkers as direct surrogates of cerebral pathophysiology. Therefore, integrating clinical metabolomics with tissue-level data from preclinical models remains essential to fully understand stroke-related metabolic dynamics and to validate candidate biomarkers mechanistically.

### The limitations of current IS metabolomics studies and future directions

Existing metabolomics studies in IS face several important limitations. Most have focused on blood or urine samples, with little exploration of alternative matrices such as saliva or feces that could offer complementary insights. Sample sizes are often small, and control groups are not always well-matched for comorbidities like hypertension or diabetes, limiting generalizability and introducing confounding effects. A key gap is the inconsistent assessment of lesion severity, a variable that can significantly influence metabolic profiles. Few studies stratify by infarct size or use standardized imaging-based measures, which hampers comparability. Methodological heterogeneity across platforms—NMR, LC-MS, GC-MS—further limits reproducibility. Differences in sample collection, processing, and data analysis contribute to batch effects and inconsistent findings. Most studies are cross-sectional and retrospective, with limited attention to prediction or causality. Few assess whether metabolomic markers can forecast recurrent stroke or long-term complications. External validation is also rare, reducing confidence in the applicability of biomarkers.

To address these gaps, future studies should adopt larger, prospectively followed cohorts with standardized protocols, careful control of confounding factors, and external validation. Integrating metabolomics with other omics—genomics, transcriptomics, proteomics—may provide a more complete understanding of IS pathophysiology and facilitate the discovery of biomarkers with true clinical utility.

## Conclusion

This study highlights the potential application value of metabolite changes and metabolic pathways in the diagnosis, differential diagnosis, risk assessment, complication identification, and prognosis of IS. However, due to the varying quality of the included studies, further validation is needed to confirm these findings.

## Data Availability

The original contributions presented in the study are included in the article/Supplementary material, further inquiries can be directed to the corresponding author.

## Glossary

AHA/ASA: American Heart Association/American Stroke Association
AIS: Acute ischemic stroke
BBB: Blood–brain barrier
BCAAs: Branched-chain amino acids
CE: Cholesterol esters
GC-MS: Gas chromatography-mass spectrometry
IS: Ischemic stroke
LC-MS: Liquid chromatography-mass spectrometry
LysoPC: Lysophosphatidylcholine
LysoPE: Lysophosphatidylethanolamine
MMP-9: Matrix metalloproteinases-9
NMR: Nuclear magnetic resonance
PAH: Phenylalanine hydroxylase
PC: Phosphatidylcholine
PG: Phosphatidylglycerol
PSD: Post-stroke depression
QUADOMICS: Quality Assessment of Diagnostic Accuracy Studies for omics
ROS: Reactive oxygen species
WHO: World Health Organization
WOS: Web of Science
