# Supplementary material for: Metabolomics reveals key biomarkers for ischemic stroke: a systematic review of emerging evidence
Source: Front Neurol. 2025 Aug 8;16:1630390. doi: 10.3389/fneur.2025.1630390 (PMC12370529; doi:10.3389/fneur.2025.1630390)
Supplement: Supplementary file 1 [file Data_Sheet_1.pdf]

Supplement 1: Search Strategy

1. Identify mesh terms and free words

2. “Metabolomics”mesh terms：metabolomics；metabolome；metabolic flux analysis；metabolic profiling；metabolic signature；metabolic biomarker；meta-bolic profile

“Metabolomics” free words ： Metabolomic; Metabonomics; Metabonomic; Metabolomes; Metabolic Profile; Metabolic Profiles; Profile, Metabolic; Profiles, Metabolic; Analysis, Metabolic Flux; Flux Analyses, Metabolic; Flux Analysis, Metabolic; Metabolic Flux Analyses

“Stroke” mesh terms：Stroke Rehabilitation；Stroke；Ischemic Stroke；Cerebral Infarction

“Stroke” free words：Rehabilitation, Stroke; Strokes; Cerebrovascular Accident; Cerebrovascular Accidents; CVA (Cerebrovascular Accident); CVAs (Cerebrovascular Accident); Cerebrovascular Apoplexy; Apoplexy, Cerebrovascular; Vascular Accident, Brain; Brain Vascular Accident; Brain Vascular Accidents; Vascular Accidents, Brain; Cerebrovascular Stroke; Cerebrovascular Strokes; Stroke, Cerebrovascular; Strokes, Cerebrovascular; Apoplexy; Cerebral Stroke; Cerebral Strokes; Stroke, Cerebral; Strokes, Cerebral; Stroke, Acute; Acute Stroke; Acute Strokes; Strokes, Acute; Cerebrovascular Accident, Acute; Acute Cerebrovascular Accident; Acute Cerebrovascular Accidents; Cerebrovascular Accidents, Acute; Ischemic Strokes; Stroke, Ischemic; Ischaemic Stroke; Ischaemic Strokes; Stroke, Ischaemic; Cryptogenic Ischemic Stroke; Cryptogenic Ischemic Strokes; Ischemic Stroke, Cryptogenic; Stroke, Cryptogenic Ischemic; Cryptogenic Stroke; Cryptogenic Strokes; Stroke, Cryptogenic; Cryptogenic Embolism Stroke; Cryptogenic Embolism Strokes; Embolism Stroke, Cryptogenic; Stroke, Cryptogenic Embolism; Wake-up Stroke; Stroke, Wake-up; Wake up Stroke; Wake-up Strokes; Acute Ischemic Stroke; Acute Ischemic Strokes; Ischemic Stroke, Acute; Stroke, Acute Ischemic; Cerebral Infarctions; Infarctions, Cerebral; Infarction, Cerebral; Cerebral Infarct; Cerebral Infarcts; Infarct, Cerebral; Infarcts, Cerebral; Cerebral Infarction, Left Hemisphere; Left Hemisphere, Infarction, Cerebral; Infarction, Left Hemisphere, Cerebral; Left Hemisphere, Cerebral Infarction; Cerebral, Left Hemisphere, Infarction; Infarction, Cerebral, Left Hemisphere; Subcortical Infarction; Infarction, Subcortical; Infarctions, Subcortical; Subcortical Infarctions; Posterior Choroidal Artery Infarction; Anterior Choroidal Artery Infarction; Cerebral Infarction, Right Hemisphere; Right Hemisphere, Cerebral Infarction; Infarction, Right Hemisphere, Cerebral; Right Hemisphere, Infarction, Cerebral; Cerebral, Right Hemisphere, Infarction; Infarction, Cerebral, Right Hemisphere.

3. Search in various databases：

3.1 Pubmed:

#1 (("Metabolome"[Mesh]) OR "Metabolomics"[Mesh]) OR "Metabolic Flux Analysis"[Mesh] 39115

#2 (((((((((((((((((((metabolomics[Text Word]) OR (metabolomicm[Text Word])) OR (metabonomics[Text Word])) OR (metabonomic[Text Word])) OR (metabolome[Text Word])) OR (metabolomes[Text Word])) OR (metabolic Profile[Text Word])) OR (metabolic profiles[Text Word])) OR (profile, metabolic[Text Word])) OR (profiles, metabolic[Text Word])) OR (Metabolic Flux Analysis[Text Word])) OR (Analyses, Metabolic Flux[Text Word])) OR (Analysis, Metabolic Flux[Text Word])) OR (Flux Analyses, Metabolic[Text Word])) OR (Flux Analysis, Metabolic[Text Word])) OR (Metabolic Flux Analyses[Text Word])) OR (metabolic profiling[Text Word])) OR (metabolic signature[Text Word])) OR (metabolic biomarker[Text Word])) OR (meta-bolic profile[Text Word]) 80851

#3 #1 OR #2 83255

#4 (((((((("Stroke Rehabilitation"[Mesh] OR "Stroke"[Mesh] OR "Ischemic Stroke"[Mesh]) OR ("Cerebral Infarction"[Mesh])) OR (((((((((((((((((((((((((Cerebral Infarctions[Text Word]) OR (Infarctions, Cerebral[Text Word])) OR (Infarction, Cerebral[Text Word])) OR (Cerebral Infarct[Text Word])) OR (Cerebral Infarcts[Text Word])) OR (Infarct, Cerebral[Text Word])) OR (Infarcts, Cerebral[Text Word])) OR (Cerebral Infarction, Left Hemisphere[Text Word])) OR (Left Hemisphere, Infarction, Cerebral[Text Word])) OR (Infarction, Left Hemisphere, Cerebral[Text Word])) OR (Left Hemisphere, Cerebral Infarction[Text Word])) OR (Cerebral, Left Hemisphere, Infarction[Text Word])) OR (Infarction, Cerebral, Left Hemisphere[Text Word])) OR (Subcortical Infarction[Text Word])) OR (Infarction, Subcortical[Text Word])) OR (Infarctions, Subcortical[Text Word])) OR (Subcortical Infarctions[Text Word])) OR (Posterior Choroidal Artery Infarction[Text Word])) OR (Anterior Choroidal Artery Infarction[Text Word])) OR (Cerebral Infarction, Right Hemisphere[Text Word])) OR (Right Hemisphere, Cerebral Infarction[Text Word])) OR (Infarction, Right Hemisphere, Cerebral[Text Word])) OR (Right Hemisphere, Infarction, Cerebral[Text Word])) OR (Cerebral, Right Hemisphere, Infarction[Text Word])) OR (Infarction, Cerebral, Right Hemisphere[Text Word]))) OR (Rehabilitation, Stroke[Text Word])) OR ((Strokes[Text Word]) OR (Cerebrovascular Accident[Text Word])) OR (Cerebrovascular Accidents[Text Word])) OR (CVA (Cerebrovascular Accident[Text Word]))) OR (CVAs (Cerebrovascular Accident[Text Word]))) OR (Cerebrovascular Apoplexy[Text Word])) OR (Apoplexy, Cerebrovascular[Text Word])) OR (Vascular Accident, Brain[Text Word])) OR (Brain Vascular Accident[Text Word])) OR (Brain Vascular Accidents[Text Word])) OR (Vascular Accidents, Brain[Text Word])) OR (Cerebrovascular Stroke[Text Word])) OR (Cerebrovascular Strokes[Text Word])) OR (Stroke, Cerebrovascular[Text Word])) OR (Strokes, Cerebrovascular[Text Word])) OR (Apoplexy[Text Word])) OR (Cerebral Stroke[Text Word])) OR (Cerebral Strokes[Text Word])) OR (Stroke, Cerebral[Text Word])) OR (Strokes, Cerebral[Text Word])) OR (Stroke, Acute[Text Word])) OR (Acute Stroke[Text Word])) OR (Acute Strokes[Text Word])) OR (Strokes, Acute[Text Word]))

OR (Cerebrovascular Accident, Acute[Text Word])) OR (Acute Cerebrovascular Accident[Text Word])) OR (Acute Cerebrovascular Accidents[Text Word])) OR (Cerebrovascular Accidents, Acute[Text Word])) OR (Ischemic Strokes[Text Word])) OR (Stroke, Ischemic[Text Word])) OR (Ischaemic Stroke[Text Word])) OR (Ischaemic Strokes[Text Word])) OR (Stroke, Ischaemic[Text Word])) OR (Cryptogenic Ischemic Stroke[Text Word])) OR (Cryptogenic Ischemic Strokes[Text Word])) OR (Ischemic Stroke, Cryptogenic[Text Word])) OR (Stroke, Cryptogenic Ischemic[Text Word])) OR (Cryptogenic Stroke[Text Word])) OR (Cryptogenic Strokes[Text Word])) OR (Stroke, Cryptogenic[Text Word])) OR (Cryptogenic Embolism Stroke[Text Word])) OR (Cryptogenic Embolism Strokes[Text Word])) OR (Embolism Stroke, Cryptogenic[Text Word])) OR (Stroke, Cryptogenic Embolism[Text Word])) OR (Wake-up Stroke[Text Word])) OR (Stroke, Wake-up[Text Word])) OR (Wake up Stroke[Text Word])) OR (Wake-up Strokes[Text Word])) OR (Acute Ischemic Stroke[Text Word])) OR (Acute Ischemic Strokes[Text Word])) OR (Ischemic Stroke, Acute[Text Word])) OR (Stroke, Acute Ischemic[Text Word]))) OR (cerebrovascular attack[Text Word])) OR (cerebral apoplexy[Text Word])) OR (acute cerebrovascular accident[Text Word])) OR (acute cerebrovascular disease[Text Word]) 22303

#5 ((review[Title]) OR (systematic review[Title])) OR (meta analysis[Title]) 815053

#6 (#3 AND #4) NOT #5 344

3.2 EMBASE:

No.	Query	Results
1	'metabolomics'/exp OR metabolomics	83240
2	'metabolome'	24393
3	'metabolic flux analysis'	2977
4	'metabolomics'	82332
5	'metabonomics'	4152
6	'metabonomic'	1789
7	'metabolic fingerprinting'	8336
8	('metabolomics'/exp OR metabolomics) OR 'metabolome' OR 'metabolic flux analysis' OR 'metabolomics' OR 'metabonomics' OR 'metabonomic' OR 'metabolic fingerprinting'	101137
9	'cerebrovascular accident'	418654
10	'stroke rehabilitation'	14633
11	'ischemic stroke'	122721
12	'brain infarction'	73637
13	stroke	597822
14	'cerebrovascular accident' OR 'stroke rehabilitation' OR 'ischemic stroke' OR 'brain infarction' OR stroke	740747
15	(('metabolomics'/exp OR metabolomics) OR 'metabolome' OR 'metabolic flux analysis' OR 'metabolomics' OR 'metabonomics' OR 'metabonomic' OR 'metabolic fingerprinting') AND ('cerebrovascular accident' OR 'stroke rehabilitation' OR 'ischemic stroke' OR 'brain infarction' OR stroke)	1139
16	'review'	6004721
17	'systematic review'	558650
18	'meta analysis (topic)'	54692
19	'review' OR 'systematic review' OR 'meta analysis (topic)'	6023109

20	((('metabolomics'/exp OR metabolomics) OR 'metabolome' OR 'metabolic flux analysis' OR 'metabolomics' OR 'metabonomics' OR 'metabonomic' OR 'metabolic fingerprinting') AND ('cerebrovascular accident' OR 'stroke rehabilitation' OR 'ischemic stroke' OR 'brain infarction' OR stroke)) NOT ('review' OR 'systematic review' OR 'meta analysis (topic)')	859
21	(((('metabolomics'/exp OR metabolomics) OR 'metabolome' OR 'metabolic flux analysis' OR 'metabolomics' OR 'metabonomics' OR 'metabonomic' OR 'metabolic fingerprinting') AND ('cerebrovascular accident' OR 'stroke rehabilitation' OR 'ischemic stroke' OR 'brain infarction' OR stroke)) NOT ('review' OR 'systematic review' OR 'meta analysis (topic)')) AND 'article'/it	582

3.3 WOS：

#	Search Query	Results
1	((((((((((TS=(metabolomics)) OR TS=(Metabolome)) OR TS=(Metabolic Flux Analysis)) OR TS=(metabolic profiling)) OR TS=(metabolic signature)) OR TS=(metabolic biomarker)) OR TS=(meta-bolic profile)) OR TS=(Metabolomic)) OR TS=(Metabonomics)) OR TS=(Metabonomic) and Preprint Citation Index (Exclude – Database)	328848
2	((((((TS=(stroke)) OR TS=(Ischemic Stroke)) OR TS=(Cerebral Infarction)) OR TS=(Cerebrovascular Accident)) OR TS=(Cerebrovascular Apoplexy)) OR TS=(Brain Vascular Accident)) OR TS=(Stroke Rehabilitation) and Preprint Citation Index (Exclude – Database)	659362
3	((TS=(review)) OR TS=(meta analysis)) OR TS=(systematic review) and Preprint Citation Index (Exclude – Database)	4486961
4	#1 AND #2 and Preprint Citation Index (Exclude – Database)	4985
5	#1 AND #2 and Preprint Citation Index (Exclude – Database)	4985
6	#4 OR #3 and Preprint Citation Index (Exclude – Database)	4491279
7	#4 NOT #3 and Preprint Citation Index (Exclude – Database)	4318
8	#4 NOT #3 and Preprint Citation Index (Exclude – Database) and Article (Document Types)	3399
9	#4 NOT #3 and Preprint Citation Index (Exclude – Database) and Article (Document Types) and Review Article (Exclude – Document Types)	3080
10	#4 NOT #3 and Preprint Citation Index (Exclude – Database) and Article (Document Types) and Review Article (Exclude – Document Types) and Other or Editorial Material or Meeting or Letter or Correction or Book or Early Access or Case Report or Abstract or Retracted Publication or Biography or News or Reference Material or Unspecified (Exclude – Document Types)	1214
11	#4 NOT #3 and Preprint Citation Index (Exclude – Database) and Article (Document Types) and Review Article (Exclude – Document Types) and Other or Editorial Material or Meeting or Letter or Correction or Book or Early Access or Case Report or Abstract or Retracted Publication or Biography or News or Reference Material or Unspecified (Exclude – Document Types) and Epidemiology or Drug Therapy or Drug Effects (Exclude – MeSH Qualifiers)	809
12	#4 NOT #3 and Preprint Citation Index (Exclude – Database) and Article (Document Types) and Review Article (Exclude – Document Types) and Other or Editorial Material or Meeting or Letter or Correction or Book or Early Access or Case Report or Abstract or Retracted Publication or Biography or News or Reference Material or Unspecified (Exclude – Document Types) and Epidemiology or Drug Therapy or Drug Effects (Exclude – MeSH Qualifiers) and Metabolism (Exclude – MeSH Qualifiers)	640
