# Supplementary material for: Metabolomics reveals key biomarkers for ischemic stroke: a systematic review of emerging evidence
Source: Front Neurol. 2025 Aug 8;16:1630390. doi: 10.3389/fneur.2025.1630390 (PMC12370529; doi:10.3389/fneur.2025.1630390)
Supplement: Supplementary file 2 [file Data_Sheet_2.pdf]

Title	Author,Date	Journal	Nation	Object of study	Gender	Group setting	Study type	Sample size	IS sampling time window	Analysis platform	Purpose of study	Sample type	Target or not	Upregulated metabolites	Down-regulated metabolites	Metabolic pathways
Metabolomic Profiles Associated With Incident Ischemic Stroke	Balasubramanian, 2022	Neurology	USA	IS	women	IS(621) HC(1294)	Multiple independent prospective cohort studies	1915	NM	LC/MS	clinical diagnosis	plasma	non-targeted	N6-Acetyllysine；methionine sulfoxide (MetSO)；sucrose/lactose/trehalose；Glucuronate		NM
Related factors based on non-targeted metabolomics methods in minor ischaemic stroke	Chen,2022a	Frontiers In Endocrinology	China	MIS	ALL	IS (400) HC (210)	case-control	610	NM	NMR	clinical diagnosis	serum	non-targeted	MISvsHCvaline, lactate, alanine, glutamate, glutamine, pyruvate, TMAO, a-glucose, b-glucose	MIS vs. HC lipids, NAG, choline, PC	TMAO metabolic pathway.
Serum Metabonomics Reveals Risk Factors in Different Periods of Cerebral Infarction in Humans	chen,2022b	Frontiers In Molecular Biosciences	China	CI	ALL	CI (143) A, time within 3 days of cerebral infarction, n first = 31, n second = 7; B, time after 3 days but within 5 days of cerebral infarction, n first = 17, n second = 8; C, time after 3 days but within 5 days of cerebral infarction, n first = 13, n second = 5; D, time after 7 days of cerebral infarction, n first = 19, n second = 11; T, cerebral infarction patients with glycosuria, n first = 20, n second = 12./healthy controls（59）; n first = 41, n second = 18.	case-control	202	after onset≤7 days	UPLC/MS-Q-TOF	clinical diagnosis	serum	non-targeted	L-Phenylalanine,Phenylacetyl-L-glutamine	phenylalanine (Phe)	3 to 7 days：Phenylalanine metabolism；3-5 days:valine, leucine,5-7：Tyrosine metabolism and phenylalanine, tyrosine, and tryptophan biosynthesis；>7 days：glycerolipid metabolism, methane metabolism, linoleic acid metabolism, and d-glutamine and d-glutamate metabolism；and isoleucine biosynthesis；0-3 days：steroid hormone biosynthesis, aminoacyl_x005f tRNA biosynthesis, taurine and hypotaurine metabolism,pyruvate metabolism, glycolysis or gluconeogenesis, primary bile acid biosynthesis, nitrogen metabolism, phenylalanine metabolism, arginine and proline metabolism, and propanoate metabolism
Urinary Metabolite Signatures for Predicting Elderly Stroke Survivors with Depression	Chen,2021	Neuropsychiatric Disease And Treatment	China	PSD	ALL	elderly stroke survivors without depression(122);elderly PSD(124); HCs(78)	case-control	324	NM	GC/MS	Complication Recognition	urine	non-targeted	palmitic acid, azelaic acid, sucrose, glyceric acid, α-aminobutyric acid, lactic acid and fructose	3-hydroxyisobutyric acid, sorbitol, indoxyl sulphate, 3-hydroxyphenylacetic acid, phenylalanine and tyrosine.	Phenylalanine, tyrosine and tryptophan biosynthesis (differential urinary metabolites: phenylalanine and tyrosine), Phenylalanine metabolism (differential urinary metabolites: phenylalanine and tyrosine), and Galactose metabolism (differential urinary metabolites: sucrose and sorbitol).
A metabonomic investigation on the biochemical perturbation in post-stroke patients with depressive disorder (PSD)	Ding,2016	Metabolic Brain Disease	China	PSD	ALL	PSD（28）PSND（27）HC（33）	case-control	88	after onset≥ 2 weeks	GC/MS	Complication Recognition	plasma	non-targeted	PSND vs control:Aspartic acid<br>PSD vsPSND:Palmitic acid;oleic acid;linoleic acid;proline; pyro-glutamate;rhamnose	PSND vs control:Palmitic acid; pyroglu-tamate; linoleic acid;phenylalanine;isoleucine; creatinine;stearic acid; proline;serine; valine; choles-terol; oleic acid	NM
Untargeted metabolomics predicts the functional outcome of ischemic stroke	Chi,2021	Journal Of The Formosan Medical Association	China taiwan	AIS	ALL	patients with favorable outcomes（77）; unfavorable outcomes（73）	prospective cohort study	150	after onset≤7 days	LC/MS	Determining Prognosis	plasma	non-targeted	NM	NM	platelet activating factor (PAF) was the compound with the strongest association with stroke outcome, which involved in proinflammatory mediators release, arachidonic acid metabolism,eosinophil degranulation, and production of reactive oxygen species
A Metabolomic Signature of Ischemic Stroke Showing Acute Oxidative and Energetic Stress	Djite,2023	Antioxidants (Basel, Switzerland)	France;Senegal	Cryptogenic IS	ALL	CS(40); had a clearly identified cause(39); HC(40)	case-control	119	recent NM	LC/MS	clinical diagnosis	plasma	targeted	Methionine sulfoxide，Acetyl carnitine，homocysteine concentrations，saturation of fatty acids	Docosahexaenoic acid，Choline	energetic metabolism, cell membrane integrity, monocarbon metabolism, and neurotransmission
Serum metabolites and risk of myocardial infarction and ischemic stroke: a targeted metabolomic approach in two German prospective cohorts	Floegel,2018	European Journal Of Epidemiology	Germa	IS; CVD	ALL	high-risk IS（268）low-risk IS（2984）	case-control	3010	ISpre-ictal	LC/MS	risk prediction	serum	targeted	Diacyl-phosphatidylcholines C38:3 ＆ C40:4; Acyl-alkyl-phosphatidylcholines C36:3, C38:3, C38:4 ＆ C40:3; 以及 Sphingomyelins C16:0, C24:0 ＆ C16:1 ＆ Hydroxy-sphingomyelin C22:1	NM	Lipid metabolism and inflammatory pathways

Amino Acid Biosignature in Plasma among Ischemic Stroke Subtypes	Goulart,2019	Biomed Research International	Brazil	IS	ALL	IS（73）HC（16）	case-control	89	after onset≥ 7 days	GC/MS	clinical diagnosis	plasma	non-targeted	IS vs.HC：Proline；Atherosclerotic thrombotic IS vs. CES + LIS:Leucine	Atherosclerotic thrombotic IS vs. CES + LIS:：Lysine; cysteine、IS vs HC ：methionine，Methionine，Alanine	TCA cycle，Folate cycle
Ischemic Stroke Progress Evaluation by 31P NMR-Based Metabonomic of Human Serum	Grandizoli,2014	Journal Of The Brazilian Chemical Society	Brazil	IS	ALL	IS（45）HC（17）	case-control	62	after onset≤7 days	NMR	clinical diagnosis	serum	non-targeted	Inorganic phosphate	Phosphatidylcholine, phosphatidylethanolamine, sphingomyelin	NM
Metabolic Profile for Prediction of Ischemic Stroke in Chinese Hypertensive Population	Guo,2019	Journal Of Stroke And Cerebrovascular Diseases	China	IS	ALL	High risk IS（66）, Low risk IS（66）	Nested case-control	132	ISpre-ictal	UPLC/MS	risk prediction	plasma	non-targeted	2-Methyl-1-Pyr-roline;7,7-dimethyl-5,8-eicosa-dienoic acid;dl-dihy-drosphingo-sine;bufexamic acid;l-thyroxine;10-hydroxy-2-decenoic acid	9-cis-Retinal；DL-Dihydrosphingosine，(2S)-OMPT; methylricinoleate; dl-indole-3-lactic acid; d-pipeco-linic acid;PC(15:0/0:0)[U]	retinol metabolism, sphingolipid metabolism, glycerophospholipid metabo_x005f lism, lysine degradation, tyrosine metabolism and trypto phan metabolism
Lipids, Lipoproteins, and Metabolites and Risk of Myocardial Infarction and Stroke	Holmes,2018	Journal Of The American College Of Cardiology	UK.et.al	IS	ALL	High risk IS（1146）, Low risk IS（1466）	Nested case-control	2612	ISpre-ictal	NMR	risk prediction	plasma	targeted	Isoleucine; leucine; glucose;acetoacetate;linoleic acid;beta hydroxybutyrate; total fatty acids;glycoprotein acetyls; omega 6 fatty acids;omega 3 fatty acids;docosahexae-noic acid;ployunsaturated fatty acids;monounsaturated fatty acids;saturated fatty acids	Cholesterol in HDL2	NM
Objective diagnosis of post-stroke depression using NMR-based plasma metabonomics	Hu,2019	Metabolic Brain Disease	China	PSD	ALL	PSD（42）; stroke patients（46）;HC（46）	case-control	134	after onset ≤ 6 months	NMR	Complication Recognition	plasma	non-targeted	PSD vsstrok patients:1-Methylhisti-dine;3-methylhisti-dine;phenylalanine IS vsHC:Glutamate; lactate; tyrosine;phenylalanine;α glucose;β-glucose	PSD vs. stroke patients:Tyrosine IS vs HC:1-Methylhistidine;3-methylhistidine;N-acetyl glycoprotein	neurotransmitter metabolism and oxidative stress
Rapid and Sensitive Differentiating Ischemic and Hemorrhagic Strokes by Dried Blood Spot Based Direct Injection Mass Spectrometry Metabolomics Analysis	Hu,2016	J Clin Lab Anal	China	CI	ALL	IS（129）;ICH（73）;HC（98）	case-control	300	after onset≤ 12 h	MS	clinical diagnosis	blood	targeted	IS vs HC :Alanine;citrulline;3-hydroxyisovaleryl carnitine IS vs ICH:Tyrosine; carnitine; leucine asparagines;proline;valine	IS vs ICH:Tiglylcarnitine	NM
UPLC-QTOF-MS-based Metabolomics Study on Ischemic Stroke Patients	Huang,2017	Chemical Journal Of Chinese Universities-Chinese	China	IS	ALL	IS（30）;HC（17）	case-control	47	after onset≤ 48 h	UPLC/MS-Q-TOF	clinical diagnosis	plasma	non-targeted	Sphinganine;phytosphingo-sine;tetradecanedioic acid;docosatrienoic acid	2-Ketobutyric acid; glutamine; pyroglutamic acid;LysoPE (0:0/22:0)	Sphingolipids; Amino acid metabolism; energy metabolism
A metabonomic approach applied to predict patients with cerebral infarction	Jiang,2011	Talanta	China	AIS	ALL	AIS（67）;HC（62）	case-control	129	after onset≤ 6 h	UPLC-TOF MS	clinical diagnosis	serum	targeted	Cysteine;oxidized glutathione;S-adenosyl-homocysteine;hydroxyeico-satetraenoic acid;hydroxyocta-decadienoic acid	Folic acid; tetrahydrofolate;adenosine; aldosterone;deoxocathasterone;betanin;sucrose 6-phosphate	efatty acid metabolism (hydroxyoctadecadienoic acid and hydroxyeicosatetraenoic acid)and steroid hormone metabolism (aldosterone and desoxycortone))
Metabolomics predicts stroke recurrence after transient ischemic attack	Jové ,2015	Neurology	USA	TIA	ALL	TIA(131)	prospective cohort study	131	after onset≤ 24 h	LC/MS	Predicting recurrence SR	plasma	non-targeted	SR vs. non-SR：Myristoyl-ethanolamine；early SR vs. late SR ：2-Hexyldecanoic acid;2-hydroxyhexa-decanoic acid;6-phospho-gluconic acid;arachi-donic acid;glutamine;l-norleucine;stearic acid;vitamin E	SR vs non-SR: 1-Monopalmitin; dode-canoic acid; meso-erythritol; threonate;LysoPC(16:0) early SR vs late SR: 10-Hydroxy capric acid;1-monopalmitin;5α-dihydroprogesterone;dl-ornithine;epineph-rine;kynurenine	NM

1H-NMR-based metabolomics study of cerebral infarction	Jung,2011	Stroke	Korea	IS	ALL	IS（81）;HC（74）	case-control	129	after onset≤9 h	NMR	clinical diagnosis	plasma,urine	non-targeted	plasma：Lactate; glyco-late; pyruvate;formate	plasma：very-low-density lipoprotein/low-density lipoprotein CH3, valine, lipid CH2CH2C_x005f_x0003_C, methanol,glutamine) urine：citrate, dimethylamine, creatinine,	anaerobic glyco_x005f lysis, folic acid deficiency
Metabolite profiling identifies a branched chain amino acid signature in acute cardioembolic stroke	Kimberly,2013	Stroke	USA	IS	ALL	IS（52）;TIA（32）	Dual-center forward-looking queue；case-control	84	after onset≤ 3 days	LC/MS	Determining Prognosis	plasma	targeted	IS vs.control：glucose；N-carbomoyl-beta-alanine	IS vs.contol：Carnitine; threonine;histidine;valine; leucine; isoleucine;methionine; glycine; proline; lysine; cysteamine;uridine;5′-adenosylhomocyst-eine;cis/trans hydroxyproline;creatinine; asparagine mRS（0-2）vs.mRS（3-6）：valine, leucine,isoleucine	NM
A metabolomic study on high-risk stroke patients determines low levels of serum lysine metabolites:a retrospective cohort study	Lee,2017	Molecular Biosystems	Korea	IS	ALL	High risk IS（161）, non-risk individuals（649）	Retrospective cohort study	810	ISpre-ictal	UPLC/MS-Q-TOF	risk prediction	serum	non-targeted	homocysteine sulfinic acid	L-Lysine；N6-acetyl-L_x005f lysine, 5-aminopentanoate, cadaverine, 2-oxoglutarate, nicotinamide, L-valine, S-(2-methylpropionyl)-dihydrolipoamide-E and ubiquinone	NM
Potential of serum metabolites for diagnosing post-stroke cognitive impairment	Liu,2015	Molecular Biosystems	China	PSCI	ALL	PSCI（30）non-PSCI（30）HC（20）	case-control	80	NM	UPLC/MS-Q-TOF	Complication Recognition	serum	non-targeted	stroke vs.HC：Carnitine; creatinine; proline; uric acid;phenylalanine;kynurenine;tyrosine;N-acetylneu-raminic acid;glutamine PSCI vs.non−PSCI：Carnitine; glutamine; uric acid; tyrosine;kynurenine;sphingosine-1-phosphate;phenylalanine	stroke vs.HC：Citric acid; valine; isoleucine;tryptophan; LysoPC（14：0）; LysoPC（18：2）; LysoPC（16：0）; LysoPC（18：1）; LysoPC（18：0）; LysoPC（20：1） PSCI vs.non−PSCI：Tryptophan; isoleucine; valine;3-indolepropionic acid;); stearoyl carnitin; palmitoylcarnitine）LysoPC（18：2）; LysoPC（16：	inflammation, neurotoxicity, bioenergetic homeo stasis, oxidative stress, and apoptosis
Discovery of Metabolite Biomarkers for Acute Ischemic Stroke Progression	Liu,2017	Journal Of Proteome Research	China	AIS	ALL	AIS（66）HC（63）	case-control	129	after onset≤9 h	GC/MS;LC/MS	clinical diagnosis	serum	non-targeted	Mannose; galactose; betaine;carnitine; acetylcarnitine）LysoPE(18:2);LysoPE(20:2); LysoPE(20:4);LysoPE(20:5)	Alanine; glycine; threonine;Isoleucine; aspartate;proline; ornithine;lysine; tricarballylic acid; serine;PI(22:2/0:0);trihydroxy palmitic acid;l-isoleucyl-l-proline；LysoPC(16:0);PA(18:3/0:0); PC(1:0/16:0);PC(5:0/5:0）	amino acid metabolism, phospholipid metabolism, fatty acid metabolism, and choline metabolism
Medium-Chain Acylcarnitines Are Associated With Cardioembolic Stroke and Stroke Recurrence	Seo,2018	Arterioscler Thromb Vasc Biol	Korea	CIS	ALL	CE patients（43）；、non-CE patients（147）；control participants（30）	case-control；Retrospective cohort study	220	after onset≤7 days	UPLC/MS	clinical diagnosis；Relapse prediction	serum	non-targeted	IS vs control participants:LysoPC(16:0);ceramide;PC(16:1);5-nitrilonorvaline;creatinine;octanoylcarnitine;Gly-L-val;decanoylcar-nitine;glutamine;sphingomyelin (16:1) CIS vs IS， SR vs non−SR：Octanoylcarnitine; decanoylcarnitine	ISvs：LysoPC(18:2); histidine;docosapentaenoic acid;glycochenodeoxycholic acid;arginine CIS vs IS:LysoPC（16：0）; LysoPC（18：0）; PC（38：7）	NM

A prospective study of serum metabolites and risk of ischemic stroke	Sun,2019a	Neurology	USA	IS	ALL	IS（114）;HC（112）;Ischemic stroke（346）;No ischemic strokeIS（3558）	nested case-control study；case-control	3904	after onset≤ 24 h	GC/MS;LC/MS	clinical diagnosis；risk prediction	serum	non-targeted	IS vs HC & HRS vs LRS:Tetradecanedioate；Hexadecanedioate	NM	NM
Potential serum biomarkers and metabonomic profiling of serum in ischemic stroke patients using UPLC/Q-TOF MS/MS	Sun,2017	Plos One	China	IS	All	IS（30）HC（30）	case-control	60	NM	UPLC/MS-Q-TOF	clinical diagnosis	serum	non-targeted	Uric acid; sphinganine;linoelaidyl carnitine;adrenoylethanolamide;PE(15:0/22:1)	LysoPE(0:0/18:3);bilirubin; LysoPC(18:2);LysoPC (16:0);PS(14:1/22:6);PC(14:0/20:4);PC(16:0/22:6)	glycerophospholipid, sphingolipid, phospho_x005f lipid, fat acid, acylcarnitine, heme, and purine metabolism
GC-MS-based metabolomics identifies an amino acid signature of acute ischemic stroke	Wang ,2017	Neurosci Letter	China	IS	ALL	AIS（40）;sex and age-matched controls（29）	case-control	69	after onset≤7 days	GC/MS	clinical diagnosis	serum	non-targeted	Lactate; tryptophan;glutamate;phenylalanine/tyrosine	Glutamate; acetamide;lactate;α-Hydroxybutyrate;sarcosine	amino acids；energy-related metabolism
Discriminating poststroke depression from stroke by nuclear magnetic resonance spectroscopy-based metabonomic analysis	Xiao,2016	Neuropsychiatric Disease And Treatment	China	PSD	ALL	PSD (94);Non-PSD (78);HC（74）	case-control	246	NM	NMR	Complication Recognition	urine	non-targeted	carnosine, arabinitol, α-glucose, phenylalanine, formate	Glutamate; acetamide;lactate;α-Hydroxybutyrate;sarcosine	1) pyruvate metabolism (lactate and formate); 2) propanoate metabolism (lactate and α-hydroxybutyrate); 3) nitrogen metabolism (phenylalanine and formate); and 4) d-glutamine and d-glutamate metabolism (α-glucose)
Lipidomic analysis of plasma in patients with lacunar infarction using normal-phase/reversed-phase two-dimensional liquid chromatography-quadrupole time-of-flight mass spectrometry	Yang,2017	Anal Bioanal Chem	China	LI	ALL	IS（35）HC（21）	case-control	56	NM	LC/MS	clinical diagnosis	plasma	non-targeted	DG（38：6）; LysoPC（20：5）; LysoPC（20：4）; LysoPC（22：6）; LysoPC（24：0）; TG（52：5）; TG（54：5）; TG（54：4）; TG（54：3）; TG（56：5）	Fatty acid (16:1); GluCer (38:2); PE (35:2)	NM
A Novel Urinary Metabolite Signature for Non-invasive Post-stroke Depression Diagnosis	Zhang,2015	Cell Biochemistry And Biophysics	China	PSD	ALL	PSD（130）;non-PSD;（128）HC（127）	case-control	385	NM	GC/MS	Complication Recognition	urine	non-targeted	azelaic acid, glyceric acid, pseudouridine, palmitic acid, sucrose, lactic acid, 5-hydroxyhexanoic acid	tyrosine, hippuric acid, phenylalanine, indoxyl sulfate,3-hydroxyisobutyric acid, b-aminoisobutyric acid, ribose,hypoxanthine, leucine, pyroglutamic acid	1) oxidative stress (azelaic acid); 2)tyrosine-phenylalanine pathway (tyrosine); 3) pheny_x005f lalanine, tyrosine, and tryptophan biosynthesis (tyrosine and phenylalanine); 4) pyrimidine metabolism (pseu douridine); 5) glycerolipid metabolism (glyceric acid)]
Distinguishing Intracerebral Hemorrhage from Acute Cerebral Infarction through Metabolomics	Zhang,2017	Rev Invest Clin	China	ACI ICH	ALL	ACI(129)ICH (128)HC(65)	case-control	322	after onset≤ 12 h	MS	clinical diagnosis	blood	non-targeted	Arginine; isovalerylcarnitine; ornithine; aspartic acid) ACI vs.ICH：Valine; threonine; citrulline; hydroxystearoylcarnitine; myristoylcarnitine; ornithine palmitoylcarnitine; lysline; leucine; methionine	ACI vsHC：Asparagine; acetylcarnitine; decanoylcarnitine; proline; beheniccarnitine; tyrosine; propionylcarnitine; valine carnitine; butyrylcarnitine; octadecanoylcarnitine; glycine; tryptophan; leucine; 3-hydroxylbutyrylcarnitine ACI vs ICH：acetylcarnitine; glutarylcarnitine; 3-hydroxylbutyrylcarnitine	NM

Metabolomic Profiles of Men and Women Ischemic Stroke Patients	Nicolas,2021	Diagnostics (Basel)	USA	AIS	ALL	AIS（36）；matched controls（36）	case-control	72	after onset≤ 24 h	UPLC/MS	clinical diagnosis	serum	non-targeted	**women：** 1-(1-enyl-palmitoyl)-2-arachidonoyl-GPC (P-16:0/20:4), 1-(1-enyl-palmitoyl)-2-palmitoyl-GPC (P-16:0/16:0), 5,6-dihydrouracil (P-16:0/20:2) **men：** 5alpha-androstan-3alpha,17beta-diol disulfate、alpha-hydroxyisocaproate、threonate、bilirubin		**women：** glycerophospholipid metabolism,pantothenate, and CoA biosynthesis, beta-alanine metabolism, linoleic acid metabolism,pyrimidine metabolism, alpha-Linolenic acid metabolism, glycerolipid metabolism, selenocompound metabolism, alanine, aspartate and glutamate metabolism, phosphatidylinositol signaling system, arachidonic acid metabolism, biosynthesis of unsaturated fatty acids,tryptophan metabolism and aminoacyl-tRNA biosynthesis) **men：** valine, leucine, and isoleucine biosynthesis, valine, leucine,and isoleucine degradation, pantothenate and CoA biosynthesis, primary bile acid biosyn_x005f_x0002_thesis, and
Metabolomic Characterization of Acute Ischemic Stroke Facilitates Metabolomic Biomarker Discovery	Qi,2022	Applied Biochemistry And Biotechnology	China	AIS	ALL	AIS（22）HC（22）	case-control	44	after onset≤ 24 h	UPLC/MS-Q-TOF	clinical diagnosis	serum	targeted	N4-Acetylcytidine;L-Tyrosine Nicotinic acid; Guanosine;lnosine	Argininosuccinic acid；β-D-Glucosamine；Glycerophosphocholine；L-Abrine；L-Pipecolic acid	aminoacyl-tRNA biosynthesis, glycerophospho_x005f lipid metabolism, lysine degradation, phenylalanine, tyrosine, and tryptophan biosynthesis metabolic pathways
Discovery of a New Biomarker Pattern for Differential Diagnosis of Acute Ischemic Stroke Using Targeted Metabolomics	Sun ,2019b	Frontiers In Neurology	China	AIS	ALL	AIS（38）vertigo（46）	case-control	84	NM	LC/MS	clinical diagnosis	blood	targeted	Arginine/ornithine (Arg/Orn) and citrulline/arginine (Cit/Arg); vaccenylcarnitine (C18:1), palmitoylcarnitine (C16), and 3-hydroxylbutyrylcarnitine (C4OH)		Arginine metabolism and carnitine metabolism
Circulating Metabolites Differentiate Acute Ischemic Stroke from Stroke Mimics	Tiedt,2020	Annals Of Neurology	Germany	IS	ALL	IS (508)；SM)（349）； HC（112）	case-control	469	after onset≤ 24 h	LC/MS	clinical diagnosis	serum	non-targeted	fatty acids, the steroids cortisol and pregnen_x005f olone sulfate, the tricarboxylic acid (TCA) cycle interme diates citrate and malate	amino acid proline, and the nucleosides adenosine, guanosine；inosine	NM
Early Warning of Ischemic Stroke Based on Atherosclerosis Index Combined With Serum Markers	Zhou,2022	Journal Of Clinical Endocrinology And Metabolism	China	IS	ALL	AI 小于4 (L120), AI 大于 4 (G120), IS AI less than 4 (SL40), and IS AI greater than 4 (SG120)	Cross-sectional studies	400	NM	UPLC/MS-Q-TOF	clinical diagnosis	serum	non-targeted	LysoPC (18:0/0:0); thiomorpholine 3-carboxylate; 2,2,2-trichloroethanol; PC (18:2/18:2); and PE-NMe (18:1/22:1)	SM (18:0/14:0), 2,4-dimethyl-1-(1-methylethyl)-benzene, 1-methylpyrrolinium, and PC (18:0/18:0)	metabolism of glycerophospholipids, and also affects the metabolism of linoleum acid, ALA metabolism, SM, ana_x005f chronic acid metabolism, and other pathways
Pivotal interplays between fecal metabolome and gut microbiome reveal functional signatures in cerebral ischemic stroke	Zhao,2022	Journal Of Translational Medicine	China	IS	ALL	IS（30）HC（30）	case-control	60	after onset≤7 days	GC/MS	clinical diagnosis	faeces；plasma；urine	non-targeted	faeces：phenylacetic acid	NM	NM
Phenylacetylglutamine, a Novel Biomarker in Acute Ischemic Stroke	Yu,2021a	Front Cardiovasc Med	China	IS	ALL	IS(901) HC（250)	case-control	1151	after onset≤ 31 days	LC/MS	clinical diagnosis	plasma	non-targeted＋targeted	phenylacetylglutamine	NM	purine metabolism, TCA cycle, steroid hormone biosynthesis, and pantothenate and oA biosynthesis
Association of Plasma Metabolic Biomarker Sphingosine-1-Phosphate With Cerebral Collateral Circulation in Acute Ischemic Stroke	Yu,2021b	Frontiers In Physiology	China	IS	ALL	IS(119：PCC/GCC(51/68)) , HC（57)	case-control	176	NM	LC/MS	clinical diagnosis	plasma	non-targeted	NM	NM	AIS vs.HC：Arginine biosynthesis，D-Glutamine and D-glutamate metabolism GCCvsPCC：Sphingolipid metabolism

Changes of Metabolites in Acute Ischemic Stroke and Its Subtypes	Wang ,2021	Frontiers In Neuroscience	China	AIS	ALL	AIS(99（LAA(49）/SAO(50）); HC(50）	case-control	154	after onset≤7 days	LC/MS	clinical diagnosis	serum	non-targeted	AISvs hc：oleic acid, linoleic acid, arachidonic acid (AA), docosahexaenoic acid (DHA), L-palmitoylcarnitine,tetradecanoylcarnitine, dodecanoylcarnitine, and decanoylcarnitine LAA vs SAO:PE 16:0	AIS vs HC: Cer (14:0), Cer (16:0), non_x005f adecanoic acid, 4-hydroxyproline, phosphatidylethanolamine (PE) (18:1), PE (18:0), propionylcarnitine, L-glutamine,L-arginine, and L-proline LAA vs SAO:L-pipecolic acid, 1-methylhistidine; PE (18:2), LysoPE (18:2), LysoPC (18:3), LysoPC (20:0), and LysoPC (18:2)	AIS vs HC:fatty acid metabolism and amino acid metabolism; LAA vs SAO: glycerophospholipid metabolism, glycosylphosphatidy linositol_x005f anchor biosynthesis, histidine metabolism, and lysine degradation
Targeted Metabolomic Profiling Reveals Association Between Altered Amino Acids and Poor Functional Recovery After Stroke	Wang ,2020	Frontiers In Neurology	China	IS	ALL	Good outcome group（20）;Poor outcome group（20）	Retrospective cohort study	40	after onset≤ 14 days	UPLC/MS	Determining Prognosis	serum	non-targeted	glutamate；arginine	Leucine-Isoleucine；Proline；Threonine	NM
Characteristic metabolic and microbial profiles in acute ischemic stroke patients with phlegm-heat pattern	Huang,2023	Journal Of Traditional Chinese Medical Sciences	China	AIS	ALL	AIS-PHP(20)； HC(20)	case-control	40	after onset≤7 days	LC/MS	clinical diagnosis	plasma	non-targeted	L-(+)-lactic acid， L-glutamic acid， L-pyroglutamic acid ，trans-cinnamic acid ， decanoic acid ， caprylic acid	5-oxo-d-proline ； 4-pyridoxate ；hypoxanthine； Inosine；guanine ；(-)-prostaglandin e2 ；(20s)-17,20-dihydroxypregn-4-en-3-one； 3-phenylpropanoic acid ； 9-oxo-10(e),12(e)-octadecadienoic acid ；tetrahydrodeoxycorticostero	amino acid metabolism, lipid metabolism, and nucleotide metabolism
Global metabolomics analysis of serum from humans at risk of thrombotic stroke	Khan,2020	Analyst	Korea	TS	ALL	TS(99)HC（301）	case-control	400	NM	LC/MS	clinical diagnosis	serum	non-targeted ＋ targeted	L-tryptophan, 3-methoxytyramine, methionine,homocysteinesulfinic acid, cysteine, isoleucine, carnitine, argi_x005f nine, linoleic acid, and sphingosine	N-acetylserotonin, serotonin, cysteine sulfinic acid, valine, lysine, and N-acetylaspartate	neuro_x005f transmitter pathway；biosynthesis of the amino acid pathway；lipid metabolism pathway； TCA cycle；purine metabolism
Predictive serum biomarkers of patients with cerebral infarction	Kong,2022	Neurological Research	China	CI	ALL	CI(28)；HC(27)	case-control	55	after onset≤7 days	UPLC/MS-Q-TOF	clinical diagnosis	serum	NM	Pipecolic acid ，L-Tyrosine， Tyramine ，Vanylglycol， L-2,4-diaminobutyric acid ， 2-Phenylglycine ，5,6-Epoxy-8,11,14-eicosatrienoic acid) potential metabolite：5,6-epoxy-8,11,14-eicosatrienoic acid, 2-phe_x005f_x0002_nylglycine, l-2,4-diaminobutyric acid, vanylglycol, tyr amine, l-tyrosine, pipecolic acid, and 5-aminoimidazole-4-carboxamide	5-Aminoimidazole-4-carboxamide ，Dimethyltryptamine ， 1-Hexadecanoyl-sn-glycero-3-phosphoethanolamine， 1-Heptadecanoyl-sn-glycero-3-phosphocholine ， Linoleic acid Palmitic acid potential metabolite：dimethyltryptamine, linoleic acid, palmitic acid, 1-hexadecanoyl-sn-glycero-3-phosphoethanola_x005f_x0002_mine, and 1-heptadecanoyl-sn-glycero-3-phospho choline	linoleic acid metabolism; pheny_x005f lalanine, tyrosine, and tryptophan biosynthesis; ▲tyr osine metabolism; AA ; metabolism; and fatty acid biosynthesis
Targeted Metabolomic Biomarkers for Stroke Subtyping	Lee,2023	Transl Stroke Res	Korea	AIS	ALL	LAA(169),CE(147), HC(30)	case-control	346	after onset≤ 3 days	LC/MS	clinical diagnosis	serum	targeted	AISvs hc :lysine and lysophosphatidylcholine acyl C16:0 LAA vs CE:lysine;Threonine;Lysophosphati_x005f dylcholine acyl 16:0	AIS vs HC：Putrescine LAA vs CE:Kynurenine Putrescine	NM
Identification of potential diagnostic biomarkers of cerebral infarction using gas chromatography-mass spectrometry and chemometrics	Li,2018	Advances	China	CI	ALL	CI(33)；HC(40)	case-control	73	NM	GC/MS	clinical diagnosis	serum	non-targeted	L-lysine, octadecanoic acid,	L-tyrosine； and lactic acid	arachidonic acid metabolism and fatty acid metabolism

Searching for Metabolic Markers of Stroke in Human Plasma via NMR Analysis	Oliveira,2023	Int J Mol Sci	Portugal	Stroke	ALL	low stroke risk LSR；(85) minior stroke risk(94)； high stroke risk(18)	case-control	112	NM	NMR	risk prediction	plasma	NM	LSR vs MSR; MSR vs HSR：asparagine;formate;creatinine; dimethyl sulfone	LSR vs MSR:isoleucine;	NM
TIA	Sidorov,2023	Journal Of Stroke And Cerebrovascular Diseases	USA	AIS	ALL	AIS(297) HC（159）	case-control	186	Acute phase: first 72h, chronic phase: 3-6 months	NMR	clinical diagnosis	serum	non-targeted		AIS vs hc:Phosphatidylcholines，Total cholines，Phosphoglycerides，Sphingomyelins;Total fatty acids， Saturated fatty acids， Monounsaturated fatty acids，Polyunsaturated fatty acids，Linoleic acid， Omega-6 fatty acids,Valine acute vs chronic：Acetoacetate，Beta-Hydroxybutyrate，Acetone;Leucine，Isoleucine， All BCAAs;Apolipoproteins(Apl B Apl Al/Apl B)	NM
Identification of a potential prognostic plasma biomarker of acute ischemic stroke via untargeted LC-MS metabolomics	Wu,2023	Proteomics Clinical Applications	China taiwan	AIS	ALL	favorable prognosis（73）;unfavourable prognosis（27）	case-control	100	after onset≤ 48 h	LC/MS	Determining Prognosis	plasma	non-targeted	Glycocholic acid		Bile acid metabolism
Identification of Potential Metabolite Markers for Middle-Aged Patients with Post-Stroke Depression Using Urine Metabolomics	Xie,2020	Neuropsychiatric Disease And Treatment	China	PSD	ALL	PSD (92)Non-PSD (89)	case-control	181	NM	GC/MS	Complication Recognition	urine	non-targeted	palmitic acid, hydroxylamine, myristic acid, glyceric acid, lactic acid, azelaic acid	tyrosine	phenylalanine, tyrosine and tryptophan biosynthesis；fatty acid biosynthesis；Glycerolipid metabolism；starch and sucrose metabolism
NMR based Serum metabolomics revealed metabolic signatures associated with oxidative stress and mitochondrial damage in brain stroke	Yadav,2023	Metab Brain Dis	India	BS	ALL	BS(108:IS/HS 48/60) HC(60)	case-control	168	after onset≤ 6 h	NMR	clinical diagnosis	serum	NM	BSvs HC:Methionine :Phenylalanine； Mannose；Glutamine； Leucine；Glutamate； Urea；Creatine；Citrate；Isoleucine；Tyrosine；Valine； Choline；3-HB ISvs HS: L-Methionine;L-Phenylalanine;DMG;2HB	BS vs HC:EQR ;Histidine ;Proline; Betaine ;Alanine ;Threonine IS vs HS:Glycine;Choline;Betaine	energy metabolism；glutamate–glutamine cycling， nitrogen metabolism.methionine metabolism
The identification of novel stroke-related sphingolipid biomarkers using UPLC-MS/MS	Yang,2024	Clinica Chimica Acta	China	Sroke	ALL	Stroke(56) HC(56)	case-control	112	NM	UPLC/MS-Q-TOF	clinical diagnosis	serum	non-targeted	ceramides, 1-phosphoceramides, phytoceramides, glycosphingolipids, sphingomyelins, and sphingomyelins		Sphingolipid metabolism

AA(arachidonic acid)，ACI (Acute Cerebral Infarction)，AI (Atherosclerosis Index)，ALA(α-linoleum acid)，BS (Brain Stroke)，CE (Cardioembolic)，CIH (Intracerebral Hemorrhage)，CIS (Cardioembolic Ischemic Stroke)，CS (Cryptogenic Stroke)，GCC (Good Collateral Circulation)，HS (Hemorrhagic Stroke)，LAA (Large Artery Atherosclerosis)，LI (Lacunar Infarction)，LIS(Lacunar Infarction stroke)，MI (Myocardial Infarction)，NM(Not Mentioned)，MIS (Minor Ischemic Stroke)，PCC (Poor Collateral Circulation)，PHP (Phlegm-Heat Pattern)，PSCI (Post-Stroke Cognitive Impairment)，PSD(Post-Stroke drepression），SAO (Small Artery Occlusion)，SR (Stroke
