# Supplementary material for: Metabolomics reveals key biomarkers for ischemic stroke: a systematic review of emerging evidence
Source: Front Neurol. 2025 Aug 8;16:1630390. doi: 10.3389/fneur.2025.1630390 (PMC12370529; doi:10.3389/fneur.2025.1630390)
Supplement: Supplementary file 3 [file Data_Sheet_3.pdf]

Table 1 Results of the QUADOMICS assessment

Reference	1	2	3	4a	4b	5	6	7	8	9	10	11	12	13	14	15	16	Score
Chen 2022b	Y	Y	Y	Y	Y	Y	Y	Y	Y	Y	Y	Y	Y	Y	NA	Y	Y	15
Chen 2021	Y	Y	Y	Y	Y	Y	Y	Y	Y	Y	Y	Y	Y	Y	NA	U	U	14
Chi 2021	Y	Y	Y	Y	Y	Y	Y	Y	Y	Y	Y	Y	Y	Y	NA	Y	Y	15
Chen 2022a	Y	Y	Y	Y	Y	Y	Y	Y	Y	Y	Y	Y	Y	Y	NA	U	U	14
Ding et al. 2016	Y	NA	Y	Y	Y	Y	Y	Y	Y	Y	Y	Y	N	Y	NA	N	Y	12
Djite,2023	Y	Y	Y	Y	Y	Y	Y	Y	Y	Y	Y	Y	N	Y	Y	N	N	13
Goulart et al. 2019	N	NA	Y	N	U	Y	Y	Y	Y	Y	Y	Y	N	Y	NA	N	Y	10.5
Grandizoli et al. 2014	N	NA	Y	Y	U	Y	Y	Y	Y	Y	Y	Y	N	Y	NA	N	N	10
Hu et al. 2016	N	NA	Y	Y	U	Y	Y	Y	Y	Y	Y	Y	N	Y	NA	N	N	10
Hu et al. 2019	Y	NA	Y	Y	Y	Y	Y	Y	Y	Y	Y	Y	N	Y	NA	N	Y	12
Huang et al. 2017	Y	NA	Y	Y	U	Y	Y	Y	Y	Y	Y	Y	N	Y	NA	N	N	11
Huang,2023	Y	Y	Y	Y	U	Y	Y	Y	Y	Y	Y	Y	Y	Y	NA	U	U	14
Jung et al. 2011	Y	NA	Y	Y	U	Y	Y	Y	Y	Y	Y	Y	N	Y	NA	N	Y	12
Jiang et al. 2011	Y	NA	Y	Y	U	Y	Y	Y	Y	Y	Y	Y	N	Y	NA	N	Y	12
Kimberly et al. 2013	Y	Y	Y	N	U	Y	Y	Y	Y	Y	Y	Y	N	Y	Y	N	Y	13.5
Khan,2020	Y	Y	Y	Y	U	Y	Y	Y	Y	Y	Y	Y	Y	Y	NA	N	N	13
Kong,2022	Y	Y	Y	Y	U	Y	Y	Y	Y	Y	Y	Y	Y	Y	Y	U	U	15
Lee,2023	Y	Y	Y	Y	U	Y	Y	Y	Y	Y	Y	Y	Y	Y	Y	U	U	15
Li,2018	Y	NA	Y	Y	U	Y	Y	Y	Y	Y	Y	Y	Y	Y	NA	U	U	13
Liu et al. 2015	Y	NA	Y	Y	U	Y	Y	Y	Y	Y	Y	Y	N	Y	NA	N	Y	12
Liu et al. 2017	Y	NA	Y	Y	U	Y	Y	Y	Y	Y	Y	Y	N	Y	NA	Y	Y	13
Nicolas 2021	Y	NA	Y	N	Y	Y	Y	Y	Y	Y	Y	Y	N	Y	Y	U	U	12.5
Qi 2022	Y	NA	Y	Y	U	Y	Y	Y	Y	Y	Y	Y	Y	Y	NA	N	Y	13

Seo et al. 2018	Y	NA	Y	N	U	Y	Y	Y	Y	Y	Y	Y	N	Y	NA	N	N	10.5
SIDOROV 2023	Y	Y	Y	Y	U	Y	Y	Y	Y	Y	Y	Y	Y	Y	NA	U	U	14
Sun et al. 2019a	Y	NA	Y	Y	U	Y	Y	Y	Y	Y	Y	Y	N	Y	NA	Y	Y	13
Sun et al. 2019b	Y	Y	Y	N	Y	Y	Y	Y	Y	Y	Y	Y	N	Y	NA	N	N	11.5
Sun et al. 2017	Y	NA	Y	Y	U	Y	Y	Y	Y	Y	Y	Y	N	Y	NA	N	Y	12
Tiedt,2020	Y	NA	Y	Y	U	Y	Y	Y	Y	Y	Y	Y	N	Y	NA	U	U	12
Wang et al. 2017	Y	NA	Y	Y	U	Y	Y	Y	Y	Y	Y	Y	N	Y	NA	N	Y	12
Wang 2021	Y	Y	Y	Y	U	Y	Y	Y	Y	Y	Y	Y	Y	Y	Y	N	U	14.5
Xiao et al. 2016	Y	NA	Y	Y	U	Y	Y	Y	Y	Y	Y	Y	N	Y	NA	N	Y	12
Xie 2020	Y	Y	Y	Y	U	Y	Y	Y	Y	Y	Y	Y	Y	Y	Y	U	U	15
Yadav 2023	Y	Y	Y	Y	U	Y	Y	Y	Y	Y	Y	Y	Y	Y	NA	U	U	14
Yang et al. 2017	Y	NA	Y	Y	U	Y	Y	Y	Y	Y	Y	Y	N	Y	NA	N	Y	12
Yang,2024	Y	Y	Y	Y	U	Y	Y	Y	Y	Y	Y	Y	Y	Y	NA	U	U	14
Yu 2021b	Y	Y	Y	Y	U	Y	Y	Y	Y	Y	Y	Y	Y	Y	NA	N	N	13
Yu 2021a	Y	Y	Y	Y	U	Y	Y	Y	Y	Y	Y	Y	Y	Y	NA	N	N	13
Zhang 2015	Y	NA	Y	Y	Y	Y	Y	Y	Y	Y	Y	Y	N	Y	NA	N	Y	12
Zhang et al. 2017	Y	NA	Y	Y	U	Y	Y	Y	Y	Y	Y	Y	N	Y	NA	N	Y	12
Zhou 2022	Y	NA	Y	Y	U	Y	Y	Y	Y	Y	Y	Y	Y	Y	NA	U	U	13
Zhao 2022	Y	NA	Y	Y	U	Y	Y	Y	Y	Y	Y	Y	Y	Y	NA	Y	Y	14

Items and scoring criteria:

1. Were selection criteria clearly described?

2. Was the spectrum of patients representative of patients who will receive the test in practice?

3. Was the type of sample fully described?

4. Were the procedures and timing of biological sample collection with respect to clinical factors described with enough detail?

4.1. Clinical and physiological factors

4.2. Diagnostic and treatment procedures.

5. Were handling and pre-analytical procedures reported in sufficient detail and similar for the whole sample? And, if differences in procedures were reported, was their effect on the results assessed?

6. Is the time period between the reference standard and the index test short enough to reasonably guarantee that the target condition did not change between the two tests?

7. Is the reference standard likely to correctly classify the target condition?

8. Did the whole sample or a random selection of the sample receive verification using a reference standard of diagnosis?

9. Did patients receive the same reference standard regardless of the result of the index test?

10. Was the execution of the index test described in sufficient detail to permit replication of the test?

11. Was the execution of the reference standard described in sufficient detail to permit its replication?

12. Were the index test results interpreted without knowledge of the results of the reference standard?

13. Were the reference standard results interpreted without knowledge of the results of the index test?

14. Were the same clinical data available when test results were interpreted as would be available when the test is used in practice?

15. Were uninterpretable/intermediate test results reported?

16. Is it likely that the presence of overfitting was avoided?

Y = meet criteria (1 point), N = does not meet criteria (0 points), U = unclear (0.5 points), NA = not applicable (0 points)

Table 2 Results of the QUIPS assessment

**Reference**	**1**	**2**	**3**	**4**	**5**	**6**	**Score**
Floegel et al. 2018	Y	Y	Y	Y	Y	Y	6
Guo et al. 2019	Y	U	Y	Y	Y	P	4.5
Holmes et al. 2018	Y	P	Y	Y	Y	P	5
Jové et al. 2015	Y	Y	Y	Y	Y	P	5.5
Kimberly et al. 2013	P	U	Y	Y	P	Y	4
Lee et al. 2017	Y	U	Y	Y	N	P	3.5
Seo et al. 2018	Y	Y	Y	Y	Y	Y	6
Sun et al. 2019a	Y	U	Y	Y	Y	Y	5
Wang 2020	Y	Y	Y	Y	Y	Y	6
Kong 2022	Y	Y	Y	Y	Y	Y	6
OLIVEIRA 2023	P	Y	Y	Y	Y	Y	5.5
Wu 2023	Y	U	Y	U	U	Y	3
Chi 2021	Y	U	Y	Y	Y	Y	5

Items and scoring criteria:

1.Study Participation：The study sample represents the population of interest on key characteristics, sufficient to limit potential bias of the observed relationship between the prognostic factor and outcome.

2.Study Attrition：Loss to follow-up is not associated with key characteristics sufficient to limit potential bias to the observed relationship between the prognostic factor and the outcome.

3.Prognostic Factor Measurement ：Prognostic factor is adequately measured in study participants to sufficiently limit potential bias.

4.Outcome Measurement：Outcome of interest is adequately measured in study participants to sufficiently limit potential bias.

5.Confounding Summary：Important potential confounders are appropriately accounted for, limiting potential bias with respect to the relationship between PF and outcome.

6.Statistical Analysis and Reporting ：The statistical analysis is appropriate for the design of the study, limiting potential for presentation of invalid or spurious results.

Y = meet standard (1 point), N = does not meet standard (0 points), P = partially meets standard (0.5 points), U = uncertain (0 points)
