# Supplementary material for: Metabolomics reveals key biomarkers for ischemic stroke: a systematic review of emerging evidence
Source: Front Neurol. 2025 Aug 8;16:1630390. doi: 10.3389/fneur.2025.1630390 (PMC12370529; doi:10.3389/fneur.2025.1630390)
Supplement: Supplementary file 4 [file Data_Sheet_4.pdf]

Supplement 5: Results of Metabolic Pathway Analysis of Potential Biomarkers for IS

Pathway Name	Match Status	*P*	-log(*p*)	Holm *p*	FDR	Impact
**IS vs. Control (clinical diagnosis)**						
Glycine, serine and threonine metabolism	9/33	6.62E-06	5.18E+00	5.29E-04	3.04E-04	4.06E-01
Valine, leucine and isoleucine biosynthesis	5/8	7.60E-06	5.12E+00	6.01E-04	3.04E-04	0.00E+00
Glyoxylate and dicarboxylate metabolism	7/32	3.51E-04	3.46E+00	2.73E-02	9.35E-03	2.17E-01
Arginine biosynthesis	4/14	2.56E-03	2.59E+00	1.97E-01	5.11E-02	3.05E-01
Phenylalanine metabolism	3/8	4.01E-03	2.40E+00	3.05E-01	6.42E-02	3.57E-01
Galactose metabolism	5/27	5.65E-03	2.25E+00	4.24E-01	7.53E-02	2.13E-01
Alanine, aspartate and glutamate metabolism	5/28	6.64E-03	2.18E+00	4.92E-01	7.59E-02	2.00E-01
Phenylalanine, tyrosine and tryptophan biosynthesis	2/4	1.10E-02	1.96E+00	8.05E-01	1.10E-01	1.00E+00
Cysteine and methionine metabolism	5/33	1.35E-02	1.87E+00	9.72E-01	1.20E-01	2.87E-01
Linoleic acid metabolism	2/5	1.79E-02	1.75E+00	1.00E+00	1.43E-01	1.00E+00
Purine metabolism	7/70	3.23E-02	1.49E+00	1.00E+00	2.16E-01	4.03E-03
Tryptophan metabolism	5/41	3.24E-02	1.49E+00	1.00E+00	2.16E-01	3.81E-01
Tyrosine metabolism	5/42	3.55E-02	1.45E+00	1.00E+00	2.18E-01	1.82E-01
Taurine and hypotaurine metabolism	2/8	4.58E-02	1.34E+00	1.00E+00	2.62E-01	0.00E+00
Sphingolipid metabolism	4/32	5.03E-02	1.30E+00	1.00E+00	2.68E-01	1.45E-01
Citrate cycle (TCA cycle)	3/20	5.57E-02	1.25E+00	1.00E+00	2.79E-01	1.81E-01
Biosynthesis of unsaturated fatty acids	4/36	7.21E-02	1.14E+00	1.00E+00	3.21E-01	0.00E+00
Arginine and proline metabolism	4/36	7.21E-02	1.14E+00	1.00E+00	3.21E-01	1.66E-01
Pyruvate metabolism	3/23	7.88E-02	1.10E+00	1.00E+00	3.32E-01	2.20E-01
Neomycin, kanamycin and gentamicin biosynthesis	1/2	8.69E-02	1.06E+00	1.00E+00	3.48E-01	0.00E+00
Glycolysis / Gluconeogenesis	3/26	1.05E-01	9.77E-01	1.00E+00	4.01E-01	1.07E-01
Glutathione metabolism	3/28	1.25E-01	9.04E-01	1.00E+00	4.54E-01	9.93E-02
Lysine degradation	3/30	1.45E-01	8.38E-01	1.00E+00	5.06E-01	0.00E+00
Histidine metabolism	2/16	1.57E-01	8.05E-01	1.00E+00	5.22E-01	2.21E-01
Starch and sucrose metabolism	2/18	1.89E-01	7.24E-01	1.00E+00	6.05E-01	4.71E-01
Glycerophospholipid metabolism	3/36	2.13E-01	6.72E-01	1.00E+00	6.34E-01	2.25E-01
Fructose and mannose metabolism	2/20	2.22E-01	6.54E-01	1.00E+00	6.34E-01	0.00E+00
Pantothenate and CoA biosynthesis	2/20	2.22E-01	6.54E-01	1.00E+00	6.34E-01	0.00E+00
Nitrogen metabolism	1/6	2.39E-01	6.21E-01	1.00E+00	6.60E-01	0.00E+00
Pyrimidine metabolism	3/39	2.49E-01	6.04E-01	1.00E+00	6.64E-01	4.54E-02
Valine, leucine and isoleucine degradation	3/40	2.61E-01	5.83E-01	1.00E+00	6.74E-01	0.00E+00
Thiamine metabolism	1/7	2.73E-01	5.64E-01	1.00E+00	6.83E-01	0.00E+00
Ascorbate and aldarate metabolism	1/9	3.37E-01	4.73E-01	1.00E+00	8.16E-01	5.24E-01

Lipoic acid metabolism	2/28	3.56E-01	4.49E-01	1.00E+00	8.37E-01	1.70E-03
Biotin metabolism	1/10	3.66E-01	4.36E-01	1.00E+00	8.37E-01	0.00E+00
Porphyrin metabolism	2/31	4.05E-01	3.93E-01	1.00E+00	8.99E-01	5.28E-02
alpha-Linolenic acid metabolism	1/13	4.48E-01	3.49E-01	1.00E+00	9.68E-01	0.00E+00
Glycosylphosphatidylinositol (GPI)-anchor biosynthesis	1/15	4.96E-01	3.05E-01	1.00E+00	1.00E+00	6.39E-03
Ubiquinone and other terpenoid-quinone biosynthesis	1/18	5.61E-01	2.51E-01	1.00E+00	1.00E+00	0.00E+00
Amino sugar and nucleotide sugar metabolism	2/42	5.66E-01	2.47E-01	1.00E+00	1.00E+00	0.00E+00
Pentose and glucuronate interconversions	1/19	5.81E-01	2.36E-01	1.00E+00	1.00E+00	9.64E-02
Arachidonic acid metabolism	2/44	5.92E-01	2.28E-01	1.00E+00	1.00E+00	1.47E-02
Primary bile acid biosynthesis	2/46	6.16E-01	2.10E-01	1.00E+00	1.00E+00	1.74E-02
beta-Alanine metabolism	1/21	6.17E-01	2.09E-01	1.00E+00	1.00E+00	0.00E+00
Propanoate metabolism	1/22	6.35E-01	1.97E-01	1.00E+00	1.00E+00	4.10E-02
Inositol phosphate metabolism	1/30	7.48E-01	1.26E-01	1.00E+00	1.00E+00	0.00E+00
Fatty acid elongation	1/39	8.34E-01	7.89E-02	1.00E+00	1.00E+00	0.00E+00
Fatty acid degradation	1/39	8.34E-01	7.89E-02	1.00E+00	1.00E+00	0.00E+00
Fatty acid biosynthesis	1/47	8.86E-01	5.27E-02	1.00E+00	1.00E+00	1.47E-02
**AIS vs. control (clinical diagnosis)**						
Glutathione metabolism	4/28	1.40E-03	2.85E+00	1.12E-01	7.00E-02	1.19E-01
Arginine biosynthesis	3/14	1.78E-03	2.75E+00	1.41E-01	7.00E-02	2.54E-01
Glycine, serine and threonine metabolism	4/33	2.62E-03	2.58E+00	2.05E-01	7.00E-02	3.11E-01
Valine, leucine and isoleucine biosynthesis	2/8	8.56E-03	2.07E+00	6.59E-01	1.71E-01	0.00E+00
Galactose metabolism	3/27	1.22E-02	1.91E+00	9.27E-01	1.95E-01	3.91E-01
Lysine degradation	3/30	1.63E-02	1.79E+00	1.00E+00	2.18E-01	0.00E+00
Arginine and proline metabolism	3/36	2.67E-02	1.57E+00	1.00E+00	3.05E-01	3.06E-01
Neomycin, kanamycin and gentamicin biosynthesis	1/2	3.65E-02	1.44E+00	1.00E+00	3.65E-01	0.00E+00
Phenylalanine, tyrosine and tryptophan biosynthesis	1/4	7.17E-02	1.14E+00	1.00E+00	6.37E-01	5.00E-01
Linoleic acid metabolism	1/5	8.88E-02	1.05E+00	1.00E+00	7.02E-01	1.00E+00
Cysteine and methionine metabolism	2/33	1.22E-01	9.15E-01	1.00E+00	7.02E-01	1.23E-01
Thiamine metabolism	1/7	1.22E-01	9.13E-01	1.00E+00	7.02E-01	0.00E+00
Purine metabolism	3/70	1.35E-01	8.70E-01	1.00E+00	7.02E-01	2.03E-02
Taurine and hypotaurine metabolism	1/8	1.38E-01	8.59E-01	1.00E+00	7.02E-01	0.00E+00
Phenylalanine metabolism	1/8	1.38E-01	8.59E-01	1.00E+00	7.02E-01	3.57E-01
Biosynthesis of unsaturated fatty acids	2/36	1.40E-01	8.52E-01	1.00E+00	7.02E-01	0.00E+00
One carbon pool by folate	1/9	1.54E-01	8.11E-01	1.00E+00	7.16E-01	0.00E+00
Valine, leucine and isoleucine degradation	2/40	1.66E-01	7.79E-01	1.00E+00	7.16E-01	0.00E+00
Biotin metabolism	1/10	1.70E-01	7.69E-01	1.00E+00	7.16E-01	0.00E+00
Butanoate metabolism	1/15	2.44E-01	6.12E-01	1.00E+00	9.77E-01	1.11E-01

Starch and sucrose metabolism	1/18	2.86E-01	5.44E-01	1.00E+00	1.00E+00	4.21E-01
Fructose and mannose metabolism	1/20	3.12E-01	5.06E-01	1.00E+00	1.00E+00	0.00E+00
Pantothenate and CoA biosynthesis	1/20	3.12E-01	5.06E-01	1.00E+00	1.00E+00	0.00E+00
Pyruvate metabolism	1/23	3.50E-01	4.56E-01	1.00E+00	1.00E+00	0.00E+00
Glycolysis / Gluconeogenesis	1/26	3.86E-01	4.14E-01	1.00E+00	1.00E+00	0.00E+00
Folate biosynthesis	1/27	3.97E-01	4.01E-01	1.00E+00	1.00E+00	0.00E+00
Lipoic acid metabolism	1/28	4.08E-01	3.89E-01	1.00E+00	1.00E+00	1.70E-03
Alanine, aspartate and glutamate metabolism	1/28	4.08E-01	3.89E-01	1.00E+00	1.00E+00	2.16E-02
Porphyrin metabolism	1/31	4.41E-01	3.56E-01	1.00E+00	1.00E+00	0.00E+00
Glyoxylate and dicarboxylate metabolism	1/32	4.52E-01	3.45E-01	1.00E+00	1.00E+00	1.06E-01
Steroid hormone biosynthesis	2/87	4.83E-01	3.16E-01	1.00E+00	1.00E+00	1.02E-02
Tyrosine metabolism	1/42	5.47E-01	2.62E-01	1.00E+00	1.00E+00	0.00E+00
Amino sugar and nucleotide sugar metabolism	1/42	5.47E-01	2.62E-01	1.00E+00	1.00E+00	0.00E+00
Primary bile acid biosynthesis	1/46	5.80E-01	2.37E-01	1.00E+00	1.00E+00	7.58E-03
**IS vs. ICH (clinical diagnosis)**						
Valine, leucine and isoleucine biosynthesis	3/8	5.67E-05	4.25E+00	4.53E-03	4.53E-03	0.00E+00
Glycine, serine and threonine metabolism	4/33	3.14E-04	3.50E+00	2.48E-02	1.26E-02	3.11E-01
Phenylalanine, tyrosine and tryptophan biosynthesis	2/4	6.50E-04	3.19E+00	5.07E-02	1.73E-02	1.00E+00
Phenylalanine metabolism	2/8	2.96E-03	2.53E+00	2.28E-01	5.91E-02	3.57E-01
Arginine biosynthesis	2/14	9.25E-03	2.03E+00	7.03E-01	1.48E-01	2.89E-01
Glutathione metabolism	2/28	3.52E-02	1.45E+00	1.00E+00	4.57E-01	8.87E-02
Lysine degradation	2/30	4.00E-02	1.40E+00	1.00E+00	4.57E-01	0.00E+00
Arginine and proline metabolism	2/36	5.57E-02	1.25E+00	1.00E+00	5.57E-01	1.81E-01
Valine, leucine and isoleucine degradation	2/40	6.73E-02	1.17E+00	1.00E+00	5.98E-01	0.00E+00
Biotin metabolism	1/10	1.03E-01	9.87E-01	1.00E+00	8.25E-01	0.00E+00
Ubiquinone and other terpenoid-quinone biosynthesis	1/18	1.78E-01	7.49E-01	1.00E+00	1.00E+00	0.00E+00
Pantothenate and CoA biosynthesis	1/20	1.96E-01	7.07E-01	1.00E+00	1.00E+00	0.00E+00
Propanoate metabolism	1/22	2.14E-01	6.70E-01	1.00E+00	1.00E+00	0.00E+00
Pentose phosphate pathway	1/23	2.22E-01	6.53E-01	1.00E+00	1.00E+00	4.42E-02
Lipoic acid metabolism	1/28	2.64E-01	5.78E-01	1.00E+00	1.00E+00	1.70E-03
Porphyrin metabolism	1/31	2.88E-01	5.41E-01	1.00E+00	1.00E+00	0.00E+00
Glyoxylate and dicarboxylate metabolism	1/32	2.96E-01	5.29E-01	1.00E+00	1.00E+00	1.06E-01
Cysteine and methionine metabolism	1/33	3.04E-01	5.18E-01	1.00E+00	1.00E+00	1.04E-01
Glycerophospholipid metabolism	1/36	3.26E-01	4.86E-01	1.00E+00	1.00E+00	2.58E-02
Fatty acid degradation	1/39	3.48E-01	4.58E-01	1.00E+00	1.00E+00	0.00E+00
Tyrosine metabolism	1/42	3.70E-01	4.32E-01	1.00E+00	1.00E+00	1.40E-01
Primary bile acid biosynthesis	1/46	3.97E-01	4.01E-01	1.00E+00	1.00E+00	7.58E-03
**PSD vs. NPSD (Complication Identification)**						
Phenylalanine, tyrosine and tryptophan	2/4	1.10E-03	2.96E+00	8.79E-02	8.79E-02	1.00E+00

biosynthesis						
Phenylalanine metabolism	2/8	4.96E-03	2.30E+00	3.92E-01	1.49E-01	3.57E-01
Galactose metabolism	3/27	5.57E-03	2.25E+00	4.35E-01	1.49E-01	3.89E-02
Biosynthesis of unsaturated fatty acids	3/36	1.25E-02	1.90E+00	9.65E-01	2.51E-01	0.00E+00
Histidine metabolism	2/16	1.99E-02	1.70E+00	1.00E+00	3.18E-01	9.02E-02
Starch and sucrose metabolism	2/18	2.49E-02	1.60E+00	1.00E+00	3.32E-01	5.48E-02
Fructose and mannose metabolism	2/20	3.04E-02	1.52E+00	1.00E+00	3.47E-01	1.31E-01
Pentose phosphate pathway	2/23	3.95E-02	1.40E+00	1.00E+00	3.95E-01	0.00E+00
Linoleic acid metabolism	1/5	6.80E-02	1.17E+00	1.00E+00	5.51E-01	1.00E+00
Glyoxylate and dicarboxylate metabolism	2/32	7.18E-02	1.14E+00	1.00E+00	5.51E-01	7.94E-02
Glycine, serine and threonine metabolism	2/33	7.58E-02	1.12E+00	1.00E+00	5.51E-01	1.20E-01
Valine, leucine and isoleucine degradation	2/40	1.06E-01	9.76E-01	1.00E+00	6.56E-01	2.71E-02
Valine, leucine and isoleucine biosynthesis	1/8	1.07E-01	9.72E-01	1.00E+00	6.56E-01	0.00E+00
Fatty acid biosynthesis	2/47	1.38E-01	8.60E-01	1.00E+00	7.89E-01	1.47E-02
Glycerolipid metabolism	1/16	2.02E-01	6.94E-01	1.00E+00	1.00E+00	9.35E-02
Ubiquinone and other terpenoid-quinone biosynthesis	1/18	2.25E-01	6.48E-01	1.00E+00	1.00E+00	0.00E+00
Pentose and glucuronate interconversions	1/19	2.36E-01	6.28E-01	1.00E+00	1.00E+00	0.00E+00
beta-Alanine metabolism	1/21	2.57E-01	5.90E-01	1.00E+00	1.00E+00	5.60E-02
Pyruvate metabolism	1/23	2.78E-01	5.56E-01	1.00E+00	1.00E+00	0.00E+00
Glycolysis / Gluconeogenesis	1/26	3.08E-01	5.11E-01	1.00E+00	1.00E+00	0.00E+00
Glutathione metabolism	1/28	3.28E-01	4.84E-01	1.00E+00	1.00E+00	7.09E-03
Arginine and proline metabolism	1/36	4.01E-01	3.97E-01	1.00E+00	1.00E+00	1.74E-02
Fatty acid elongation	1/39	4.26E-01	3.70E-01	1.00E+00	1.00E+00	0.00E+00
Fatty acid degradation	1/39	4.26E-01	3.70E-01	1.00E+00	1.00E+00	0.00E+00
Amino sugar and nucleotide sugar metabolism	1/42	4.50E-01	3.46E-01	1.00E+00	1.00E+00	0.00E+00
Tyrosine metabolism	1/42	4.50E-01	3.46E-01	1.00E+00	1.00E+00	1.40E-01
Purine metabolism	1/70	6.35E-01	1.97E-01	1.00E+00	1.00E+00	1.62E-02
**PSCI vs. NPSCI (Complication Identification)**						
Phenylalanine, tyrosine and tryptophan biosynthesis	2/4	2.64E-04	3.58E+00	2.11E-02	2.11E-02	1.00E+00
Valine, leucine and isoleucine biosynthesis	2/8	1.21E-03	2.92E+00	9.59E-02	3.24E-02	0.00E+00
Phenylalanine metabolism	2/8	1.21E-03	2.92E+00	9.59E-02	3.24E-02	3.57E-01
Valine, leucine and isoleucine degradation	2/40	2.99E-02	1.52E+00	1.00E+00	5.02E-01	0.00E+00
Tryptophan metabolism	2/41	3.13E-02	1.50E+00	1.00E+00	5.02E-01	2.37E-01
Nitrogen metabolism	1/6	4.12E-02	1.38E+00	1.00E+00	5.50E-01	0.00E+00
Purine metabolism	2/70	8.27E-02	1.08E+00	1.00E+00	9.38E-01	0.00E+00
Arginine biosynthesis	1/14	9.38E-02	1.03E+00	1.00E+00	9.38E-01	0.00E+00
Ubiquinone and other terpenoid-quinone biosynthesis	1/18	1.19E-01	9.24E-01	1.00E+00	1.00E+00	0.00E+00
Pantothenate and CoA biosynthesis	1/20	1.32E-01	8.81E-01	1.00E+00	1.00E+00	0.00E+00
Alanine, aspartate and glutamate	1/28	1.80E-01	7.46E-01	1.00E+00	1.00E+00	1.14E-01

metabolism						
Lysine degradation	1/30	1.91E-01	7.18E-01	1.00E+00	1.00E+00	0.00E+00
Glyoxylate and dicarboxylate metabolism	1/32	2.03E-01	6.93E-01	1.00E+00	1.00E+00	0.00E+00
Sphingolipid metabolism	1/32	2.03E-01	6.93E-01	1.00E+00	1.00E+00	1.56E-02
Fatty acid degradation	1/39	2.42E-01	6.17E-01	1.00E+00	1.00E+00	0.00E+00
Pyrimidine metabolism	1/39	2.42E-01	6.17E-01	1.00E+00	1.00E+00	0.00E+00
Tyrosine metabolism	1/42	2.58E-01	5.89E-01	1.00E+00	1.00E+00	1.40E-01
**Recurrence vs. non-recurrence（Complication Identification）**						
Biosynthesis of unsaturated fatty acids	2/36	1.30E-02	1.88E+00	1.00E+00	1.00E+00	0.00E+00
Nitrogen metabolism	1/6	3.01E-02	1.52E+00	1.00E+00	1.00E+00	0.00E+00
Arginine biosynthesis	1/14	6.91E-02	1.16E+00	1.00E+00	1.00E+00	0.00E+00
Pentose phosphate pathway	1/23	1.11E-01	9.54E-01	1.00E+00	1.00E+00	1.12E-01
Alanine, aspartate and glutamate metabolism	1/28	1.34E-01	8.73E-01	1.00E+00	1.00E+00	1.14E-01
Glyoxylate and dicarboxylate metabolism	1/32	1.52E-01	8.19E-01	1.00E+00	1.00E+00	0.00E+00
Pyrimidine metabolism	1/39	1.82E-01	7.40E-01	1.00E+00	1.00E+00	0.00E+00
Tryptophan metabolism	1/41	1.91E-01	7.20E-01	1.00E+00	1.00E+00	9.42E-02
Tyrosine metabolism	1/42	1.95E-01	7.10E-01	1.00E+00	1.00E+00	1.17E-02
Arachidonic acid metabolism	1/44	2.03E-01	6.92E-01	1.00E+00	1.00E+00	2.77E-01
Fatty acid biosynthesis	1/47	2.16E-01	6.66E-01	1.00E+00	1.00E+00	0.00E+00
Purine metabolism	1/70	3.05E-01	5.15E-01	1.00E+00	1.00E+00	0.00E+00
Steroid hormone biosynthesis	1/87	3.66E-01	4.37E-01	1.00E+00	1.00E+00	1.37E-02
**High risk vs. Low risk (risk forecast)**						
Valine, leucine and isoleucine biosynthesis	3/8	3.05E-05	4.52E+00	2.44E-03	2.44E-03	0.00E+00
Valine, leucine and isoleucine degradation	4/40	2.98E-04	3.53E+00	2.35E-02	1.19E-02	0.00E+00
Butanoate metabolism	2/15	7.21E-03	2.14E+00	5.63E-01	1.92E-01	1.11E-01
Neomycin, kanamycin and gentamicin biosynthesis	1/2	1.77E-02	1.75E+00	1.00E+00	3.54E-01	0.00E+00
Alanine, aspartate and glutamate metabolism	2/28	2.43E-02	1.61E+00	1.00E+00	3.89E-01	4.81E-02
Biosynthesis of unsaturated fatty acids	2/36	3.89E-02	1.41E+00	1.00E+00	5.00E-01	0.00E+00
Linoleic acid metabolism	1/5	4.37E-02	1.36E+00	1.00E+00	5.00E-01	1.00E+00
Biotin metabolism	1/10	8.57E-02	1.07E+00	1.00E+00	8.57E-01	0.00E+00
Arginine biosynthesis	1/14	1.18E-01	9.28E-01	1.00E+00	1.00E+00	0.00E+00
Nicotinate and nicotinamide metabolism	1/15	1.26E-01	9.00E-01	1.00E+00	1.00E+00	1.94E-01
Starch and sucrose metabolism	1/18	1.49E-01	8.26E-01	1.00E+00	1.00E+00	4.21E-01
Pantothenate and CoA biosynthesis	1/20	1.64E-01	7.84E-01	1.00E+00	1.00E+00	0.00E+00
Citrate cycle (TCA cycle)	1/20	1.64E-01	7.84E-01	1.00E+00	1.00E+00	5.86E-02
Galactose metabolism	1/27	2.16E-01	6.66E-01	1.00E+00	1.00E+00	3.50E-02
Glutathione metabolism	1/28	2.23E-01	6.52E-01	1.00E+00	1.00E+00	0.00E+00
Lipoic acid metabolism	1/28	2.23E-01	6.52E-01	1.00E+00	1.00E+00	0.00E+00
Lysine degradation	1/30	2.37E-01	6.25E-01	1.00E+00	1.00E+00	0.00E+00
Glyoxylate and dicarboxylate metabolism	1/32	2.51E-01	6.01E-01	1.00E+00	1.00E+00	0.00E+00

Tyrosine metabolism	1/42	3.16E-01	5.00E-01	1.00E+00	1.00E+00	0.00E+00
Metabolism of xenobiotics by cytochrome P450	1/68	4.62E-01	3.35E-01	1.00E+00	1.00E+00	1.02E-02
**Good outcome vs. poor outcome (outcome prediction)**						
Valine, leucine and isoleucine biosynthesis	4/8	4.09E-09	8.39E+00	3.28E-07	3.28E-07	0.00E+00
Valine, leucine and isoleucine degradation	3/40	2.88E-04	3.54E+00	2.28E-02	1.15E-02	0.00E+00
Arginine and proline metabolism	2/36	7.19E-03	2.14E+00	5.61E-01	1.92E-01	1.42E-01
Arginine biosynthesis	1/14	5.22E-02	1.28E+00	1.00E+00	1.00E+00	7.61E-02
Pantothenate and CoA biosynthesis	1/20	7.39E-02	1.13E+00	1.00E+00	1.00E+00	0.00E+00
Glycine, serine and threonine metabolism	1/33	1.19E-01	9.23E-01	1.00E+00	1.00E+00	0.00E+00
